# Leaching of dissolved organic carbon from mineral soils plays a significant role in the terrestrial carbon balance

**DOI:** 10.1111/gcb.15460

**Published:** 2020-12-14

**Authors:** Mahdi Nakhavali, Ronny Lauerwald, Pierre Regnier, Bertrand Guenet, Sarah Chadburn, Pierre Friedlingstein

**Affiliations:** ^1^ College of Life and Environmental Sciences University of Exeter Exeter UK; ^2^ Biogeochemistry and Modelling of the Earth System Department Geoscience, Environment and Society Université Libre de Bruxelles Bruxelles Belgium; ^3^ Université Paris‐Saclay INRAE AgroParisTech UMR ECOSYS Thiverval‐Grignon France; ^4^ Laboratoire de Géologie de l'ENS PSL Research University Paris France; ^5^ College of Engineering, Mathematics and Physical Sciences University of Exeter Exeter UK; ^6^ Laboratoire de Meteorologie Dynamique Departement de Geosciences Institut Pierre‐Simon Laplace CNRS‐ENS‐UPMC‐X Ecole Normale Superieure Paris France

**Keywords:** dissolved organic carbon, global terrestrial carbon, leaching, mineral soils, terrestrial carbon balance

## Abstract

The leaching of dissolved organic carbon (DOC) from soils to the river network is an overlooked component of the terrestrial soil C budget. Measurements of DOC concentrations in soil, runoff and drainage are scarce and their spatial distribution highly skewed towards industrialized countries. The contribution of terrestrial DOC leaching to the global‐scale C balance of terrestrial ecosystems thus remains poorly constrained. Here, using a process based, integrative, modelling approach to upscale from existing observations, we estimate a global terrestrial DOC leaching flux of 0.28 ± 0.07 Gt C year^−1^ which is conservative, as it only includes the contribution of mineral soils. Our results suggest that globally about 15% of the terrestrial Net Ecosystem Productivity (NEP, calculated as the difference between Net Primary Production and soil respiration) is exported to aquatic systems as leached DOC. In the tropical rainforest, the leached fraction of terrestrial NEP even reaches 22%. Furthermore, we simulated spatial‐temporal trends in DOC leaching from soil to the river networks from 1860 to 2010. We estimated a global increase in terrestrial DOC inputs to river network of 35 Tg C year^−1^ (14%) from 1860 to 2010. Despite their low global contribution to the DOC leaching flux, boreal regions have the highest relative increase (28%) while tropics have the lowest relative increase (9%) over the historical period (1860s compared to 2000s). The results from our observationally constrained model approach demonstrate that DOC leaching is a significant flux in the terrestrial C budget at regional and global scales.

## Graphical abstract

Using a process‐based, integrative, modelling approach to upscale from existing observations, we estimate a global terrestrial DOC leaching flux of 0.28 ± 0.07 Gt C year^−1^ which is conservative, as it only includes the contribution of mineral soils. Our results suggest that globally about 15% of the terrestrial Net Ecosystem Productivity is exported to aquatic systems as leached DOC. In the tropical rainforest, the leached fraction of terrestrial NEP even reaches 22%.

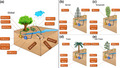

## 1 INTRODUCTION

Earth System Models (ESMs) are process‐based models that represent the full climate system including feedbacks with the carbon (C) cycle (Friedlingstein et al., 2006). They are key tools to predict climate change in response to anthropogenic perturbations and are an important input to the regular IPCC report (Eyring et al., 2016). Nevertheless, some key mechanisms are still missing in most of those models, including lateral fluxes of C from the land to ocean. It has been hypothesized that the exclusion of lateral C transfers in land surface components of ESMs implies a significant overestimation of soil heterotrophic respiration and/or C accumulation in global C budget accounting (Ciais et al., 2020; Jackson et al., 2002; Janssens et al., 2003). With state‐of‐the‐art ESMs such as those used for the 5th assessment report of the IPCC (Ciais et al., 2013), the non‐representation of lateral C transfers may thus induce a biased quantification of the land C sink and its response to changing CO_2_ and climate (Janssens et al., 2003; Walsh et al., 2017).

The net ecosystem productivity (NEP) corresponds to the net natural C exchange between terrestrial ecosystems and the atmosphere, and is traditionally defined as the difference between net primary production (NPP) and soil heterotrophic respiration (SHR). However, in this definition, NEP does not account for the lateral C exports (LCE) from terrestrial ecosystems to the inland water network. Three important contributors to the LCE are leaching of dissolved organic C (DOC) and dissolved inorganic C (DIC), and erosion of particulate organic C (POC). The leaching of DOC from soils (DOC_LCE_) which originates mainly from root exudates and the decomposition of plant residues and humus (Kalbitz et al., 2000; Khomutova et al., 2000; Van den berg et al., 2012) contributes about 37% of the global riverine C exports to the coast (Meybeck, 1993). Soil DOC is an important source of C for soil microorganisms (Kalbitz et al., 2000) and a part of mineral soil C sequestration, transport and stabilization mechanism (Neff & Asner, 2001; Sanderman & Amundson, 2008). Its transfer from soils to the inland water network through runoff and drainage is an important process in the assessment of terrestrial C budgets (Kindler et al., 2011).

However, DOC_LCE_ remains poorly constrained to date at global scale. A direct quantitative assessment of DOC leaching through the terrestrial‐aquatic ecosystems interface is currently critically missing due to the scarcity of direct observations. Previous studies have thus used fluvial DOC export to the ocean as a surrogate for DOC_LCE_ (Schlesinger & Melack, 1981). Empirical approaches can indeed predict fluvial DOC fluxes from a variety of allochthonous sources in the river catchment (Harrison et al., 2005; Lauerwald et al., 2012; Ludwig et al., 1996; Worrall et al., 2012). However, such approaches implicitly assume that DOC behaves as a conservative tracer in aquatic systems, whereas it is an important substrate for microorganisms living in the rivers (Fischer et al., 2002) and losses of DOC in transit can thus, be significant (e.g. Battin et al., 2009). In addition, phytoplankton (Descy et al., 2002) and submerged litter decomposition (Lauerwald et al., 2017) can be important in‐stream sources of DOC. Thus, estimating DOC_LCE_ from an aquatic perspective is complex because of losses and additions during transit through the inland water network (Lauerwald et al., 2012). Arguably, a more reasonable way to assess global DOC_LCE_ and its spatio‐temporal variations is to rely on a process‐based modelling approach of DOC leaching, taking advantage of the limited global DOC data set for model calibration or evaluation. Such process‐based model can then be used at the global scale, to quantify the production and cycling of DOC within the soil column as well as the subsequent DOC leaching fluxes from terrestrial soils to inland waters (Lauerwald et al., 2017).

Although several models have been developed and tested successfully to simulate DOC dynamics at site level to regional scales (Bowring et al., 2019; Hastie et al., 2019; Kicklighter et al., 2013; Lauerwald et al., 2017; Michalzik et al., 2003; Ren et al., 2016; Smith et al., 2007; Tian et al., 2015), none of these models have so far addressed soil DOC cycling at the global scale and its sensitivity to large‐scale spatial patterns in climate, vegetation and soil properties. Here we use the recently upgraded Joint UK Land Environment Simulator (Nakhavali et al., 2018) to simulate the global distribution of soil DOC stocks and leaching fluxes from terrestrial to aquatic ecosystems. More specifically, the objectives of this paper were to: (i) calibrate the model for global‐scale applications, using a large collection of site data across different ecosystems; (ii) apply the model to provide the first global estimate of soil DOC stocks and fluxes for present‐day conditions; (iii) analyse the dominant spatial patterns in stocks and fluxes and their key environmental drivers; and (iv) assess the contribution of soil DOC processes to the regional and global terrestrial C balance.

## 2 MATERIALS AND METHODS

### 2.1 Data for model parameterization and evaluation

**FIGURE 1:**
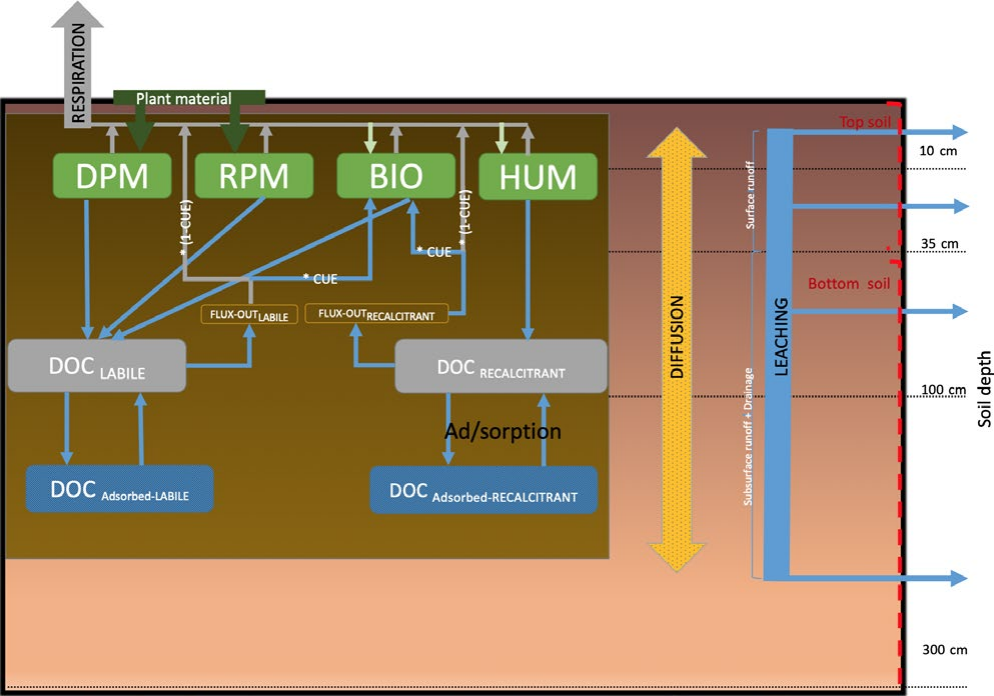
JULES‐DOCM model structure, representing C and dissolved organic carbon pools and fluxes at four soil layers

JULES‐DOCM was previously calibrated and evaluated at five temperate sites (Nakhavali et al., 2018). To constrain JULES‐DOCM with observations at the global scale, we compiled the largest global data set of measured soil DOC concentrations for the recent period (1980–2010). We only retained locations containing at least two measurements (*n* = 109). These data were then partitioned according to the different ecosystem types (Figure 1a; Table S1). All surface soil layer DOC measurements falling within one model grid‐cell (1.25° latitude, 1.875° longitude) were aggregated and the resulting grid‐cell average observed DOC concentration (*n* = 38) was then used for model parameterization (Section 2.2). To complement our analysis of soil DOC, we further confronted the model results against observed DOC concentration in sub‐surface soil layers when available.

In addition to the DOC concentration in soils, we also used observed DOC concentration in headwater streams to evaluate simulated DOC leaching fluxes, DOC_LCE_. We retained only small rivers in the analysis, in which the aquatic DOC concentrations should be closely related to that of in the soil runoff. Furthermore, we used the measured DOC leaching flux regionalized using Costal Segmentation and related Catchments (COSCAT) scheme (Meybeck et al., 2006). The global land surface is subdivided into 15 COSCAT regions representing groups of river basins which are tributary to the same coastline segment. (Seitzinger et al., 2005).

### 2.2 JULES‐DOCM

**FIGURE 2:**
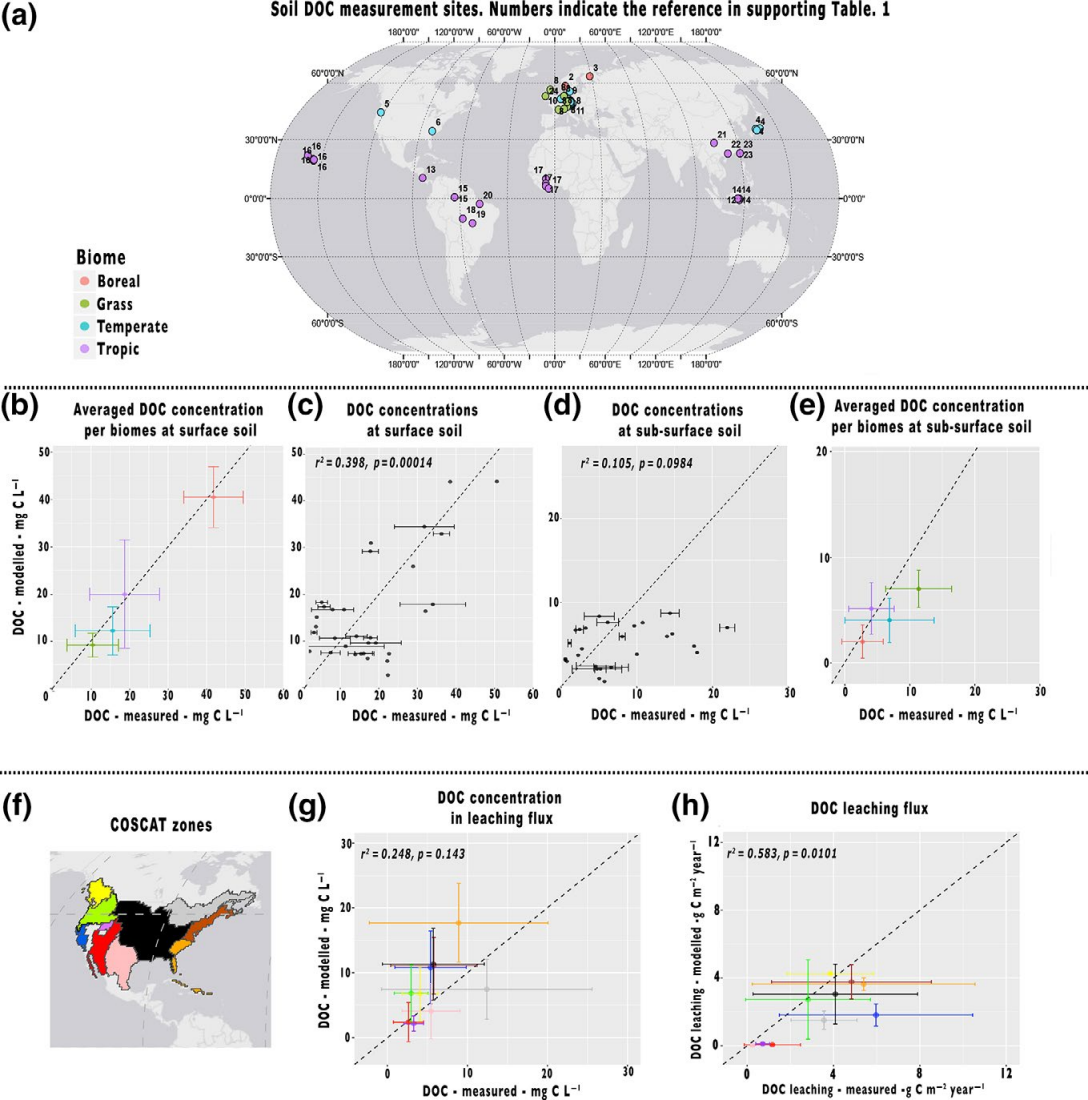
Model validation. (a) soil dissolved organic carbon (DOC) measured sites; (b) averaged DOC concentration per biome at surface soil; (c) and (d) individual measured vs. modelled soil DOC concentration at surface and sub‐surface soil; (e) averaged DOC concentration per biome at sub‐surface soil; (f) COSCAT zones; (g) measured DOC concentration; and (h) leaching fluxes in headwater streams against simulated values aggregated at the scale of the COSCAT regions across the United States (map bottom left; Meybeck et al., 2006). Colours on the plots correspond to those indicated on the map

JULES‐DOCM (JULES‐Dissolved Organic Carbon Model; Figure 2) is based on JULES, the land surface component of the UK‐ESM (Sellar et al., 2020). JULES represents the vegetation dynamics for nine distinct plant functional types (PFTs; Harper et al., 2016) and the C budgets of vegetation biomass and soil column, thus allowing for the quantification of gross primary productivity (GPP), NPP, soil respiration (Rh), NEP or net biome productivity (NBP) in response to climate, atmospheric CO_2_ and land use changes (LUC). In order to briefly assess the overall performance of the model, we compared simulated global GPP and NPP from JULES‐DOCM against data from the model tree ensemble (MTE) GPP (Jung et al., 2011) and the MODIS‐17 NPP (Zhao et al., 2005). We also compared simulated and measured soil organic carbon (SOC) concentrations for all grid‐cells for which DOC measurements were available (see below). Note that where measured SOC data were not reported, we compared simulated SOC concentrations against values reported in the Harmonized World Soil Database (HWSD) (Nachtergaele et al., 2010). Importantly, the nine PFTs used in JULES‐DOCM only cover terra firme ecosystems and exclude peatlands. Due to model limitations, our estimation of DOC_LCE_ focuses on mineral soils only, excluding peatlands and organic rich soils, which together represent about 3% of the total land area (Leifeld & Menichetti, 2018).

In JULES, SOC processes are represented following the RothC model (Jenkinson et al., 1990), which distinguishes four C pools (*n*) with different turnover times (Coleman & Jenkinson, 2014), which are two plant litter pools (DPM: decomposable plant material and RPM: resistant plant material) and two soil C pools (BIO: biomass and HUM: humus). Soil DOC cycling is simulated for each model grid‐cell over a 3 m soil profile vertically discretized into four soil layers (*i*), including the production associated with SOC and litter decomposition, and losses by biological consumption and leaching (Clark et al., 2011). In JULES‐DOCM, we modified the RothC scheme and distributed the simulated SOC pools vertically over four soil layers (from top to bottom: 0–10, 10–35, 35–100 and 100–300 cm), assuming an exponential decay of SOC with depth (Koven et al., 2013). The e‐folding parameter for this decay is based on the C content decrease at the depth relative to the surface and it is derived from SOC profiles for different biomes (Jobbágy & Jackson, 2000).

The sources of DOC originate from decomposition of the four C pools, SOC (BIO and HUM pools) and litter (DPM and RPM pools; Equation 1), a process that is controlled by soil moisture, temperature, vegetation cover and soil texture (clay and silt content). These environmental factors are collectively referred to as ‘rate modifier’ in Equation (1) and the DOC production (in each soil layer (*i*) and pool (*n*)) in our model is thus represented as:

(1) $$F_{p_{n,i}}=S_{c_{n}}\times \left(1‐e^{\left(‐K_{P}\times {\text{ratemodifiers}}_{i}\right)}\right),$$

where $S_{c_{n}}$ is the SOC content in the soil and *K*_p_ is the basal DOC production rate which depends on the C pool source.

Two DOC pools are distinguished, depending on whether they are derived from labile or recalcitrant SOC and litter pools. These DOC pools then decompose at a rate controlled by their respective reactivity and the temperature (Clark et al., 2011) in each soil layer (Equation 2). A fraction of the decomposed DOC, defined by the carbon use efficiency (CUE; Kalbitz et al., 2000; Manzoni et al., 2012), returns to the SOC pool while the remainder is released to the atmosphere as CO_2_ (Figure 2). DOC decomposition ($F_{D_{n,i}}$) thus reads:

(2) $$F_{D_{n,i}}=S_{{\text{DOC}}_{n,i}}\times \left(1‐e^{\left(‐K_{{\text{DOC}}_{n}}\times {\text{FT}}_{i}\right)}\right),$$

where $S_{{\text{DOC}}_{n,i}}$ stands for the DOC content in soil, $K_{{\text{DOC}}_{n}}$ is the basal decomposition rate of DOC and FT*_i_* is a rate modifier that only depends on temperature in each soil layer.

Each of the two DOC pools can exchange between a dissolved and an adsorbed phase, and the equilibrium exchange coefficients for adsorption depend on soil texture and pH (Moore et al., 1992). In addition, the model represents the vertical transport of dissolved DOC, including diffusion and advective leaching. Diffusion between layers depends on the DOC concentration gradient and the molecular diffusion coefficient for DOC (Ota et al., 2013). DOC leaching ($F_{{\text{DOC}}_{{\text{LCE}}_{i}}}$) is diagnosed from soil DOC concentrations and simulated runoff and drainage (${\text{Roff}}_{i},$ note that Roff for the surface soil (*i* = 1,2) leaching is surface runoff and for sub‐surface leaching (*i* = 3,4) is the sum of sub‐surface runoff and drainage) and soil moisture at each soil layer ($T_{S_{i}}$):

(3) $$F_{{\text{DOC}}_{{\text{LCE}}_{i}}}=S_{{\text{DOC}}_{n,i}}\times \frac{{\text{Roff}}_{i}}{T_{S_{i}}}.$$

The soil DOC concentration (mg C L^−1^) is diagnosed from soil DOC stocks (g C m^−2^) and soil water contents of each soil layer. The concentration in surface runoff is assumed to be from the top 35 cm and the concentration in sub‐surface runoff and drainage is based on the rest of the soil column. Adsorbed DOC is assumed inert and immobile. All these processes are calculated for each soil layer and at a 30‐min time step. A full description of the model is available in Nakhavali et al. (2018).

Studies have shown that vegetation controls the quality and concentration of DOC in the soil (Kalbitz et al., 2000) as well as the DOC leaching out of the soil (Kindler et al., 2011; Lauerwald et al., 2012). Hence the DOC decomposition and leaching rate are affected by the dominant vegetation type(s) which can be related to the lignin and polyphenol content in the soil (Kalbitz et al., 2000). In JULES, the vegetation of each grid‐cell is represented in the model as an ensemble of different PFTs of which each is assigned an areal proportion (Harper et al., 2016). In this revised version of JULES‐DOCM, we calibrated the base rates of DOC production (*K*_p_) and decomposition (*K*_DOC_) dependent on the dominant PFT per grid‐cell. Given that the model is not sensitive to the labile DOC residence time, that is, the inverse of *K*_DOC_ (Nakhavali et al., 2018), we calibrated only the *K*_DOC_ of the recalcitrant DOC pool on a per PFT basis, using a range of published estimates to bound possible values for this parameter (Table 1). As *K*_p_ values have not been reported in the literature, we used a range of values between 1 and 2 (day).

**TABLE 1:** *K*_DOC_ literature values, *K*_p_ and *K*_DOC_ default and optimized values at four main biomes

	*K*_DOC_ literature^a^ (day)	*K*_p_ default (day)	*K*_DOC_ default (day)	*K*_p_ optimized (day)	*K*_DOC_ optimized (day)
Boreal forest	820 4545	1	600	1.24	3284
Temperate forest	602 2127	1	600	1	611
Tropical subtropical forest	609 819	1	600	1.9	636
Grassland and cropland	500 1000	1	600	1.2	601

^a^ Range of *K*_DOC_ based on biodegradability of plant materials from Kalbitz et al. (2003), Yule and Gomez (2009) and Johnson et al. (2000).

The calibration of JULES‐DOCM (*K*_p_ and *K*_DOC_) was based on observed surface soil DOC concentrations (0–35 cm) only, because data density is significantly higher for shallow soils and sampling depths are more consistently defined across the globe for this layer. Moreover, most of the DOC is generally located in the surface soil layer (see e.g. Guggenberger & Kaiser, 2003). Observed subsoil (>35 cm) DOC concentrations were only used for model validation. Furthermore, because of limited data coverage, the nine PFTs of JULES‐DOCM were aggregated into four broad biomes: boreal, temperate, subtropical and tropical, and grassland and croplands (Figure S7). Next, for each of these four biomes, the kinetic parameters for DOC production and decomposition rates were calibrated through an optimization procedure minimizing the mismatch between observed and modelled DOC concentrations. Note that each site for which observed surface soil DOC concentration was available was assigned to a single PFT, based on the dominant land cover type reported for this specific location.

To select the PFT‐dependent *K*_p_ and *K*_DOC_ values that best fit observed soil DOC concentrations, we applied the Latin hypercube method to extract random pairs of *K*_p_ and *K*_DOC_ values within their bounded domains. This method requires selection of a number of random samples at least 10 times larger than the number of variables to be tested. In this work, we thus extracted, for each biome, 25 random combinations of *K*_p_ and *K*_DOC_ values within the observed ranges. We also performed a cross validation using the *K* fold method (Refaeilzadeh et al., 2009). To do so, the pool of observed surface soil DOC concentrations for each class of biome was split between a calibration and a validation set. Based on each pair of *K*_p_ and *K*_DOC_ values, we calculated the average RMSE for both calibration and validation sites. As the RMSE results from the various cross validation sets did not show a significant difference, we used all the surface soil DOC observations per biome for our final parameter calibration, choosing the combination of *K*_p_ and *K*_DOC_ which gave the lowest RMSE. The best pair of *K*_p_ and *K*_DOC_ values that were retained for each biome is reported in Table 1. These PFT‐dependent kinetic parameters for soil DOC production and decomposition were then used to calculate DOC stocks and fluxes globally at the spatial resolution of JULES‐DOCM.

River DOC concentrations in low‐order streams are a good integrator of the soil DOC leached in the draining catchments (Kicklighter et al., 2013), hence we also compared DOC concentrations in the runoff simulated by the calibrated model with observed riverine DOC concentrations for a densely surveyed region that covered different biomes: the United states, for which 623 measurements were extracted from the GloRiCh database (Hartmann et al., 2014). Where instantaneous discharge measurements were also available in GloRiCh, we evaluated the modelled DOC leaching fluxes as well. For the evaluation, the simulated values against observed were aggregated at the scale of the coastal segmentation and related catchments (COSCAT) regions across the United States (Meybeck et al., 2006).

### 2.3 Boundary conditions and forcings

JULES model requires nine meteorological driving variables, downward components of shortwave and longwave radiation at the surface, rainfall, snowfall, wind speed and direction, atmospheric surface temperature, specific humidity and pressure at 30‐min time step. Moreover, soil texture, atmospheric CO_2_ concentration and land cover changes are required as well (Best et al., 2011). Here we used meteorological data from CRU‐NCEP version 4 (Harris et al., 2014), observed atmospheric CO_2_ (Dlugokencky & Tans, 2013) and land cover change for cropland from HYDE v 3.1 (Klein Goldewijk et al., 2011). The vegetation cover was prescribed using European Space Agency Land Cover Climate Change Initiative (ESA LC_CCI) global vegetation distribution (Harper et al., 2016; Poulter et al., 2015).

As modelling of biogeochemical cycles is dependent on the initial values, these values should be provided either using observation or model values at steady state. In order to reach the steady state, a long spin‐up simulation using representative climate data repeated over a defined steady‐state period is required until there is no trend for changes in simulated pool sizes. In JULES, as the SOC needs several thousand years to reach a steady state, in particular in higher latitudes, we used an accelerated spin‐up method (Thornton & Rosenbloom, 2005), which only requires 200–300 years of spin‐up, following the simulation protocol by Harper et al. (2016). However, our simulated SOC should be lower than that simulated by the standard version of JULES due to the decomposition of SOC to DOC. In the model, the four different pools have different decomposition rates (3.2e‐7, 9.6e‐9, 2.1e‐8 and 6.4e‐10). This method scales up each pool decomposition rates to the fastest labile pool, that is, multiplying the resistant plant material, soil biomass and humus decomposition rate by 33, 15, 500 respectively. The model is then run for 200–300 years until all these scaled pools reach equilibrium. Then each pools size is multiplied by these scaling factors. The transient run for the period 1860–2010 was then performed using the final model results from the spin‐up runs. In order to analyse the long‐term trends, we calculated the 10‐year running means of the simulations result to suppress year‐to‐year fluctuations which would blur the picture.

### 2.4 DOC_LCE_ and the terrestrial C balance

To quantify the relative importance of C loss from leaching on the terrestrial C balance, we used NEP (estimated from JULES as NPP‐Rh) as a measure of net C uptake as this is the natural component of the land C balance. The export ratio is hence estimated as DOC_LCE_/NEP. We suggest not to use NBP (Equation 4) usually defined as the difference between Net Ecosystem Exchange (NEE, Equation 5) and the C flux associated with anthropogenic land use changes (E_LUC_). NBP is a C source in regions where deforestation is dominant, making the DOC_LCE_/NBP ratio negative and would hence be meaningless:

(4) $$\text{NBP}=\text{NEE}‐E_{\text{LUC}},$$

(5) NEE=‐NEP.

## 3 RESULTS AND DISCUSSION

### 3.1 Evaluation of the model for growth primary production, net primary production and soil organic carbon

Our average global estimate of GPP for the simulation period amounts to 127 Pg C year^−1^, which is only slightly higher than the MTE value of 118 Pg C year^−1^ (±5% range of the observational product; Jung et al., 2009; Figure S2a). The simulated average global NPP amounts to 88 Pg C year^−1^ which is somewhat higher than the MODIS‐17 estimate of 54 Pg C year^−1^ (±17% range of the observational product; Figure S2b). In contrast, our SOC stock amounts to 1015 Pg C which is lower than the estimate from HWSD of 1263 Pg C (Figure S2c). SOC underestimation is due to too high SOC decomposition. Given the model overestimation of terrestrial NPP, lower than observed litter input is unlikely, at least at the global scale. Hence, too high soil C decomposition (Sitch et al., 2015) is the more likely reason for the SOC underestimation, calling for a better parameterization of SOC decomposition rates in the model. Furthermore, better representation of soil C cycling will decrease the possible biases introduced to the DOC module due to under/overestimated SOC. In particular, upgrading JULES‐DOCM to the vertically resolved version of soil SOC as recently done in a different version of JULES (Burke et al., 2017) will in future improve the results. Additionally, our model does not represent peatlands, which could also contribute to the underestimation of SOC stocks (about 20%) compared to the HWSD.

### 3.2 Soil DOC stocks and leaching

#### 3.2.1 Model evaluation

The surface soil DOC concentration and averaged RMSE is 33 ± 3 mg C L^−1^ for boreal forests, 15.5 ± 11 mg C L^−1^ for temperate forests, 5.4 ± 9 mg C L^−1^ for subtropical/tropical forests and 9.4 ± 7 mg C L^−1^ for grass/croplands. The RMSEs reported here for subtropical/tropical forests and grass/croplands are not significantly different from those using the initial parameterization from Nakhavali et al. (2018), which was obtained with prescribed PFTs (a single PFT for each site). However, the recalibration markedly reduces the absolute number of all model residuals for these two biomes. For temperate forests, the average RMSE is always slightly higher than the one using the parameters values of Nakhavali et al. (2018). This can be explained by the fact that the model was initially developed using sites from temperate ecosystems only (Carlow [grassland], Braaschaat [temperate forest], Hainich [temperate forest], Turkey Point 89 [temperate forest] and Guandaushi [grassland]). Therefore, the parameter set values of Nakhavali et al. (2018) already gives the best results. However, our database mostly includes temperate forests measurements with a wide range of different characteristics, which can explain the slightly higher RMSE for this ecosystem compared to the others. Lastly, for boreal forests, the optimization of simulation performance is reflected by a substantial decrease in the averaged RMSE as a result of higher DOC resident time and DOC production. Therefore, we used the optimized parameter values for boreal forest.

Figure 1b–e represents the correlation between modelled and observed DOC concentrations for surface and sub‐surface soil, per site (Figure 1c,e) and aggregated per biome (Figure 1b,d). For the surface soil (Figure 1b,c), there is a significant (*p* < 0.001, *R* ^2^ = 0.398) correlation between observed and modelled DOC concentrations at individual sites. The correlation obtained for results aggregated per biome is even higher (*p* < 0.001, *R* ^2^ = 0.91). For sub‐surface soil (Figure 1d,e), the calibration yields a slightly more significant correlation between modelled and observed DOC concentrations (*R* ^2^ = 0.13, *p* = 0.108) compared to the one using the parameters values of Nakhavali et al. (2018; *R* ^2^ = 0.08, *p* = 0.209). However, due to the significantly smaller range of DOC measurements from sub‐surface soil compared to the surface soil, a more precise calibration is not possible. Moreover, the correlation for results aggregated per biome is higher (*p* < 0.001, *R* ^2^ = 0.49), despite the relatively narrow range of observations compared to surface soil. Hence, the vast majority of the differences between biomes comes from surface soils. Lastly, since the tropical zone was less well covered by observations and produces some of the highest fluxes (see below), we evaluated the model in more detail at the available tropical sites, which indicate a reasonable model performance (see Table S6).

The model could overestimate the DOC degradation by ignoring the increasingly recalcitrant OC with depth (Catalán et al., 2016; Sanderman & Amundson, 2008). The developments of JULES‐DOCM were based on the single‐layered and non‐discretized SOC version of JULES (vn4.4). For the simulation of DOC production and DOC cycling in the soil, for which we use vertically discretized scheme with four soil layers, we estimated the vertical distribution of each soil SOC and litter pool over these four layers assuming an exponential decay with depth using biome dependent scaling factors (Nakhavali et al., 2018). Hence, integrating the vertically discretized SOC scheme embedded in the latest version of JULES (Burke et al., 2017) into JULES‐DOCM would be the next step in model development and could help to resolve this overestimation. Moreover, there is in general a higher uncertainty in the deep soil measurements compared to surface soil, as sub‐surface soil concentrations are controlled mostly by adsorption while surface soil concentrations are controlled mainly by biodegradation (Kalbitz et al., 2000; Michalzik et al., 2001; Sanderman & Amundson, 2008).

The simulated DOC concentrations in the headwaters (Figure 1f) and associated leaching fluxes are overall in fair agreement with observed fluvial DOC exports (Figure 1g,h, *R* ^2^ of 0.24 *p* = 0.143, for concentrations and *R* ^2^ of 0.58 *p* = 0.0101 for leaching). Although we retained only small rivers in the analysis, in which the DOC concentrations should be closely related to that of the runoff, such comparison is not straightforward as model results do not account for potentially important in‐stream sources of DOC by, for example, litter decomposition, or sinks of DOC by decomposition of more labile DOC fraction in the headwaters which have possibly occurred in some of the measured sites and reflected in the measurements. Simulations also ignore the distinct DOC dynamics in saturated soils along the riparian zone, which may hamper an accurate reproduction of river DOC fluxes.

In contrast to the DOC concentrations in headwaters, our simulated DOC leaching fluxes (the runoff component of DOC_LCE_) are generally lower than the measured ones. This is not surprising as the hydrological component of JULES, previously evaluated (Gedney & Cox, 2003) and applied at global scale (Gedney et al., 2006) simulates lower runoff compared to measured discharges for the COSCAT located in the United States (Figure S4).

#### 3.2.2 Spatial patterns in soil DOC stocks and fluxes

The annual globally simulated soil DOC concentration averages to 29.3 ± 2.4 mg C L^−1^ in the surface soil, and 8.3 ± 1.4 mg C L^−1^ in the sub‐surface soil, all reported range being based on a sensitivity analysis (see Section 3.4 below). Temperate and tropical biomes exhibit the highest soil surface soil DOC concentrations (40.5 and 40 mg C L^−1^ respectively) as already reported in previous studies (Dalva & Moore, 1991; Neff & Asner, 2001) followed by subtropical and boreal biomes (19 and 18 mg C L^−1^ respectively). However, tropical biomes show the highest sub‐surface soil DOC concentration (13 mg C L^−1^) while the temperate biome exhibits the lowest sub‐surface soil concentration (4 mg C L^−1^). The mean DOC stocks per unit surface area are highest in temperate regions (3.5 g C m^−2^), intermediate in the tropics (2.9 g C m^−2^) and lowest in the boreal (2.1 g C m^−2^) and subtropical (1.1 g C m^−2^) biomes.

**FIGURE 3:**
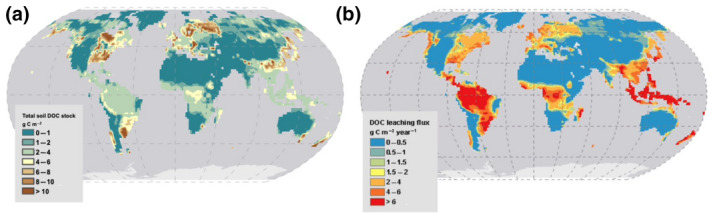
Simulated soil dissolved organic carbon (DOC) stock (g C m^−2^; left panel) and soil DOC leaching flux (g C m^−2^ year^−1^; right panel). Surface and sub‐surface soil DOC concentration in Figure S8

The spatial patterns in simulated soil DOC stocks (Figure 3a) are significantly correlated with those of SOC stocks (Figure S5a), as further evidenced by the statistics reported in the scatterplots shown in Figure S6. However, this correlation has only a *R* ^2^ of 0.48, which indicates that other factors also play a role in determining the global distribution of soil DOC stocks in the model.

To better understand the partial spatial decoupling between SOC and DOC stocks, we computed three time constants from our model results, which are related to depth‐integrated DOC production rate (*K*_prod_ = DOC production/SOC stocks), DOC decomposition rate (*K*_dec_ = DOC decomposition/DOC stocks) and DOC leaching rate (*K*_leach_ = DOC leaching/DOC stocks). The DOC production (Equation 1) is controlled by temperature and moisture while temperature is the only climatic driver of the decomposition (Equation 2), runoff being the main climatic driver of the leaching flux (Equation 3). Assuming a near steady‐state system, the DOC stock at each grid point can be diagnosed as the product of the SOC stock by *K*_prod_/(*K*_leach_ + *K*_dec_) and the simulated residence time (τDOC) of DOC in the soil column is given by *τ*DOC = 1/(*K*_dec_ + *K*_leach_).

**FIGURE 4:**
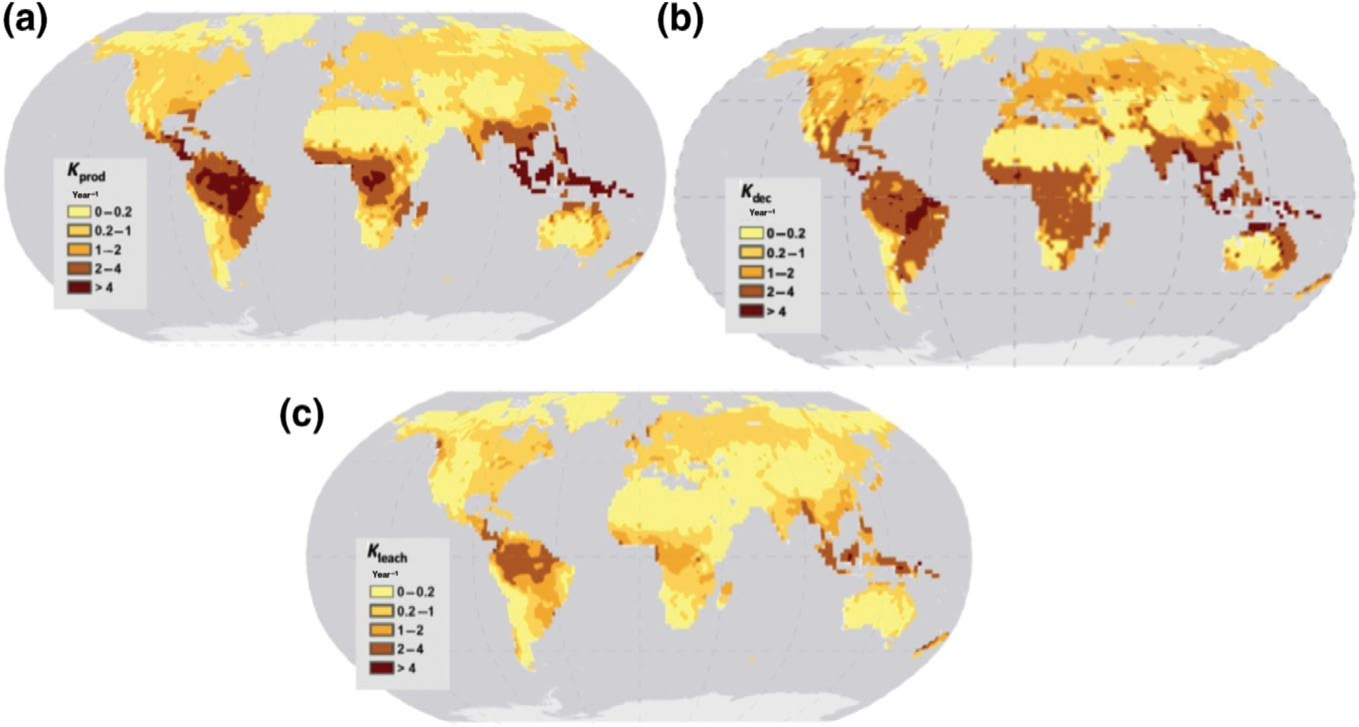
Simulated present‐day (a) production rate (*K*_prod_; year^−1^), (b) decomposition rate (*K*_dec_; year^−1^) and (c) leaching rate (*K*_leach_; year^−1^)

Altogether, the first‐order latitudinal pattern in DOC stock (Figure 3a) results from the partly opposing effects of SOC stocks that are highest in boreal and temperate regions and lowest in the (sub)tropics (Figure S7a) and of the ratio *K*_prod_/(*K*_leach_ + *K*_dec_), which is highest in the (sub)tropics and lower in the other biomes (Figure S7b). This explains why the increasing latitudinal gradient in SOC stock from tropical to boreal biomes is not as prominent for DOC stocks. In terms of time constants, *K*_prod_ has largest values in tropical regions, which is due to both moisture and temperature having a positive effect on the DOC production rate (Figure 4a), and, overall, this variable increases by at least one order of magnitude from high to low latitudes. *K*_dec_, and to a lesser extent *K*_leach_, shows a similar pattern but less prominent in quantitative terms (Figure 4b,c). Nevertheless, the higher *K*_dec_ and *K*_leach_ in tropical soils lead to a simulated DOC residence time in the soil column (*τ*DOC) of only a few weeks compared to more than a year in the boreal region (Figure S7a), a result which is in line with previous studies (Johnson et al., 2000; Kalbitz et al., 2003; Yule & Gomez, 2009).

**FIGURE 5:**
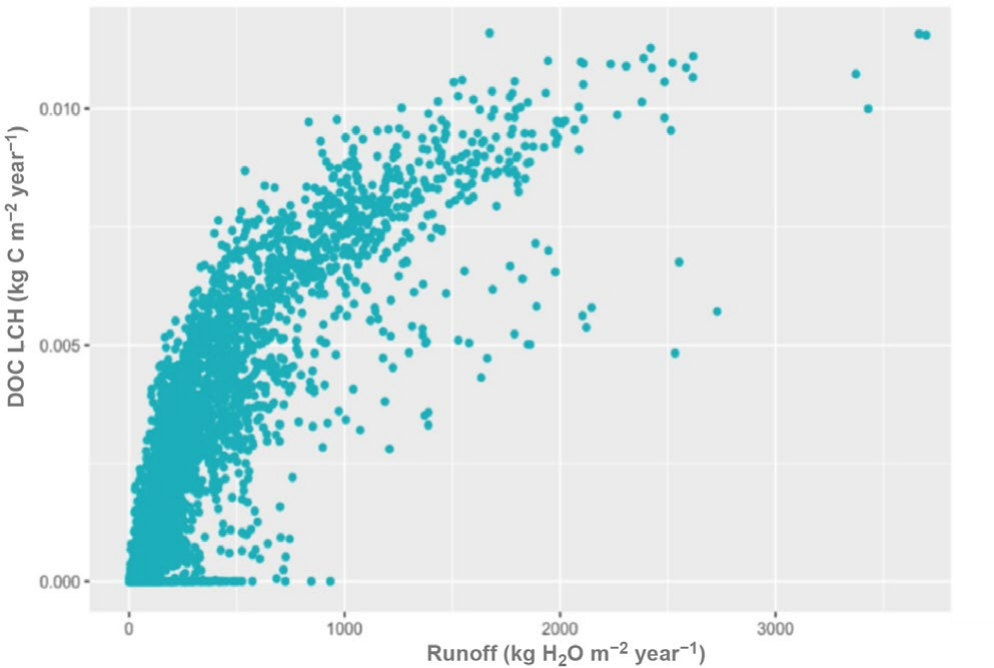
Dissolved organic carbon leaching flux (kg C m^−2^ year^−1^) against runoff (kg H_2_O m^−2^ year^−1^)

**FIGURE 6:**
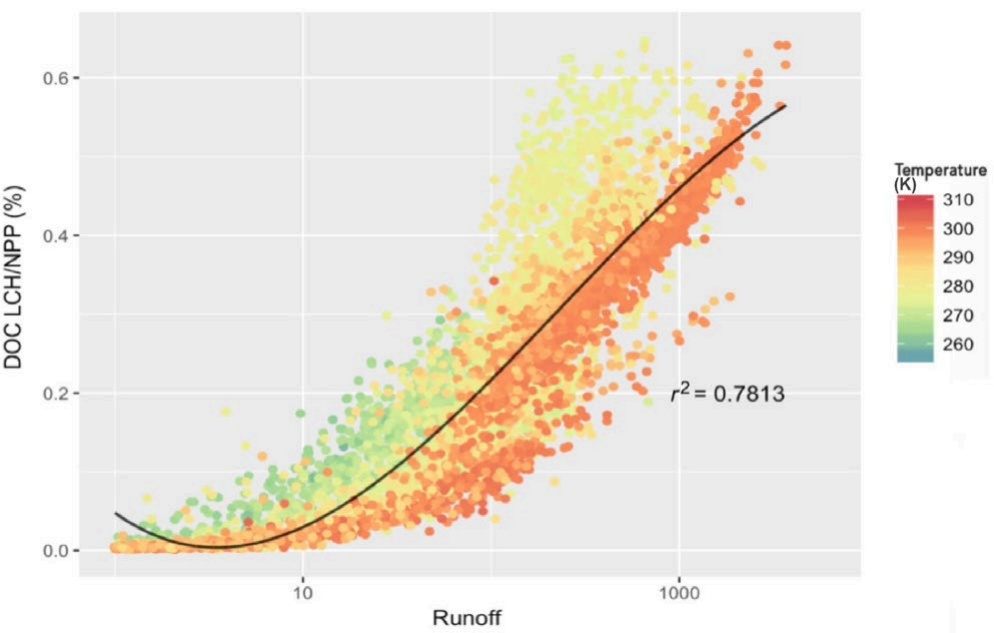
Dissolved organic carbon leaching to terrestrial net primary production ratio (%) against runoff (kg H_2_O m^−2^ year^−1^) and temperature (K). Note the log scale for the *X*‐axis

The spatial distribution of the DOC leaching flux, DOC_LCE_ (Figure 3b), which is the product of the water flux times DOC stocks, is to a large extent controlled by the distribution of runoff, and hence precipitation, across the globe (Figure S5b). Closer inspection nevertheless reveals a non‐linear behaviour, with a steady increase in DOC leaching at low to intermediate runoff values, followed by a lower rate of increase at high runoff, when available DOC stocks becomes a limiting factor for leaching (Figure 5). Limited DOC stocks are a result of the higher DOC decomposition rates in soil due to the high temperature in the tropics. This pattern is typical for a transition from transport‐limited to substrate‐limited behaviour (Figure 6).

Despite these effects, the leaching fluxes per unit area are overall higher in the tropics, the generally lower DOC stocks in this latitudinal band being largely compensated by the much higher runoff. Regionally, areas on land that show the highest DOC_LCE_ include SE Asia, New Zealand and a small portion of the South American continent where both drivers (runoff and DOC stocks) are high (Figure 3a; Figure S5). Other hotspot areas include the Amazon, and to a lesser extent, the Congo basins, as well as Western Europe and large portions of the Eastern part of North America, the latter two regions revealing intermediate runoff values, but high DOC stocks.

### 3.3 DOC leaching in the terrestrial C budget

**FIGURE 7:**
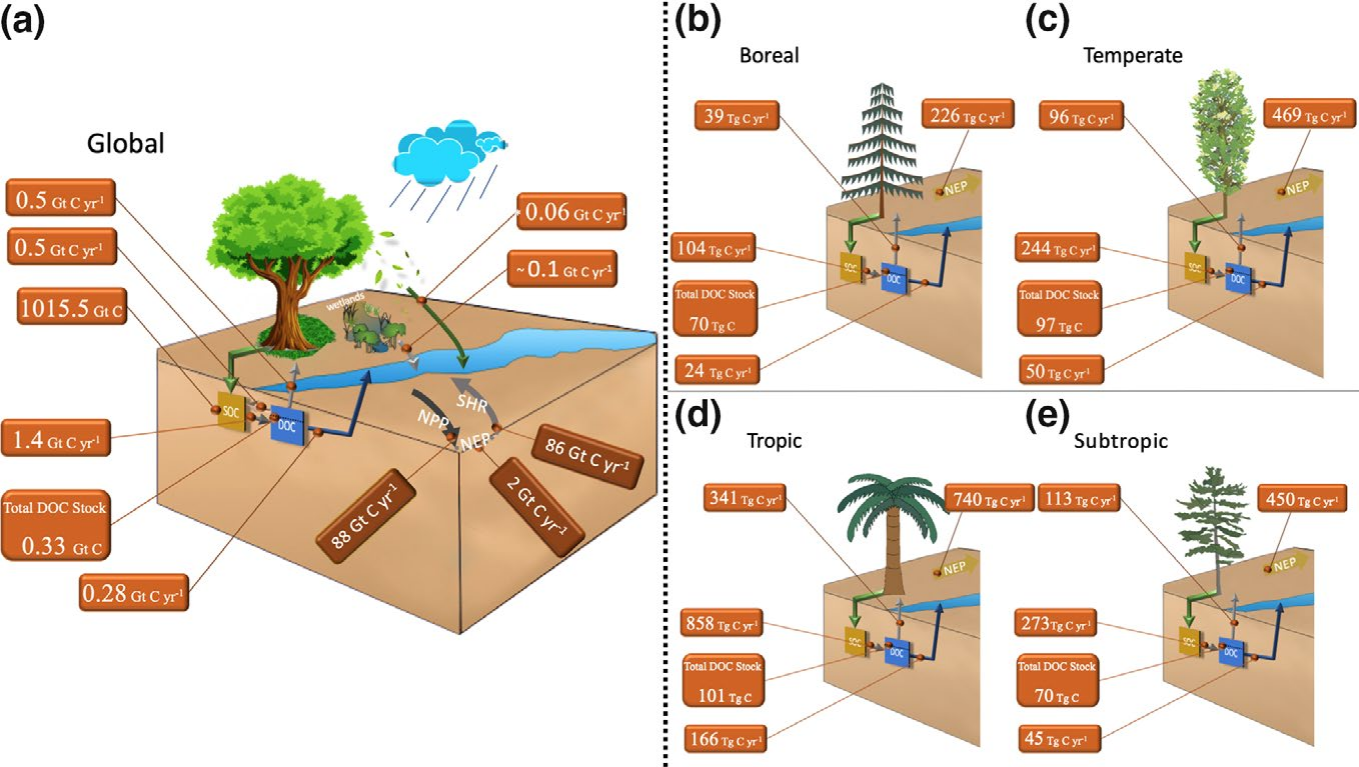
(a) Global and (b–e) regional C and dissolved organic carbon stocks and fluxes. Units are Gt C and Gt C year^−1^ for global, and Tg C and Tg C year^−1^ for regional estimates. Values from Table 2 and Table S2

Globally, we estimate the production of DOC from litter and SOC decomposition in mineral soils to be approximately 1.4 ± 0.1 Gt C year^−1^, of which 40% (0.5 ± 0.03 Gt C year^−1^) is decomposed in the soil and released back as CO_2_ to the atmosphere, 40% is transformed back into SOC, and about 20% is leached to aquatic systems (Figure 7a). Our global estimate of DOC leaching directly originating from mineral soils is thus equal to 0.28 ± 0.07 Gt C year^−1^ (DOC_LCE_, Figure 7a). The present‐day global DOC stock stored in soils is estimated at 0.30 ± 0.04 Gt C, of which 30% is concentrated in the top 10 cm. DOC production is highest for the tropics (858 ± 15.4 Tg C year^−1^), followed by the subtropical (273 ± 19 Tg C year^−1^), temperate (244 ± 26 Tg C year^−1^) and boreal (104 ± 14.8 Tg C year^−1^) regions (Figure 7b–e). The same decreasing order is simulated for the DOC mineralization fluxes while for leaching fluxes about 60% occurs in the tropical band, a result in agreement with the very high river CO_2_ emission rate in this region (Lauerwald et al., 2015). Consistent with these, the total soil DOC stocks are highest in the tropical region (101 ± 6 Tg C), followed by the temperate (97 ± 11.6 Tg C), boreal (70 ± 10.3 Tg C) and subtropical (70 ± 9.3 Tg C) biomes. The globally averaged residence time of soil DOC is remarkably short, only of the order of 4 months. DOC leaching in temperate areas is slightly higher than in the subtropics and is lowest in the boreal region. Despite much smaller absolute fluxes, the boreal region exhibits a slightly higher export to production ratio (23%) than the subtropics (16%) and tropics (19%) due to the highest residence time and lowest decomposition rate in the boreal biome.

Our global‐scale DOC_LCE_ from mineral soils is estimated at 0.28 ± 0.07 Gt C year^−1^. There are two additional potentially significant terrestrial sources of DOC that can possibly contribute to the leached DOC fluxes which should be considered to give a global DOC export estimate. First, plant litter fall can directly support in‐stream DOC production from litter decomposition. As a first‐order assessment, we use the litter production simulated by JULES and an estimate of the fractional global areal coverage of streams and rivers (Lauerwald et al., 2015; Naipal et al., 2018), and quantify the DOC production flux from litter decomposition at 0.12 Gt C year^−1^. Assuming a CUE of 0.5 (Manzoni et al., 2012), half of this decomposed litter material would directly be oxidized to CO_2_ while the remainder, about 0.06 Gt C year^−1^, would be transformed into DOC feeding in the global river network (Figure 7a), bringing our global estimate to 0.34 ± 0.07 Gt C year^−1^. Second, JULES does not account for the soil biogeochemistry associated with wetlands, which represent an estimated C stock and C accumulation rate of 110–455 Gt C and 45–210 Tg C (Botch et al., 1995) respectively. Previous model estimates from GlobalNEWS‐2 suggest that wetlands could contribute up to about 20% of fluvial DOC export to the coastal ocean (Harrison et al., 2005; Mayorga et al., 2010). Assuming that this ratio also holds for DOC exported from land to aquatic systems, and DOC from all terrestrial sources have similar reactivity within aquatic systems, we would estimate a global wetland DOC flux into inland waters of less than 0.1 Gt C year^−1^ as a very first‐order assessment. Therefore, the inclusion of estimates of DOC inputs from litter fall and wetlands leads to a total global DOC leaching flux from terrestrial ecosystems to aquatic systems of the order of 0.4–0.5 Gt C year^−1^.

**FIGURE 8:**
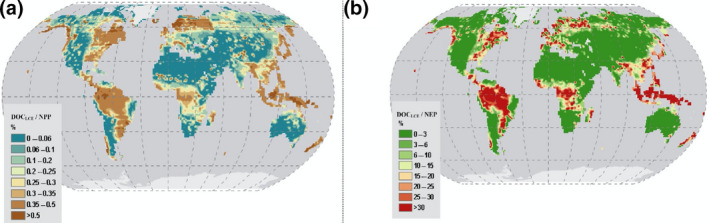
Ratio of dissolved organic carbon leaching fluxes to (a) terrestrial net primary production (%) and (b) terrestrial net ecosystem productivity (%)

The ratio of DOC_LCE_ to the terrestrial productivity (NPP; Figure 8a) is generally low and rarely exceeds 0.5%. This C export to NPP ratio (0.38%) is lower than previous global estimates (e.g. Regnier et al., 2013). This is to be expected as our estimate only includes the DOC export from mineral soils while Regnier et al. (2013) provide an estimate of total C export (in all forms) from the land. However, focusing now on the global terrestrial C budget, we estimate the DOC_LCE_ /NEP ratio, which represent the fraction of the terrestrial C sink (driven by atmospheric CO_2_ increase and climate change) that is actually lost from terrestrial systems and exported to the aquatic environment. The export ratio (DOC_LCE_/NEP) is significant and reaches almost a total of 15% at the global scale, revealing that DOC leaching is a significant term in the assessment of terrestrial C stock changes. The spatial patterns (Figure 8b) in this export ratio also reveal much higher values in the tropics compared to other regions (see also Table 2). Moreover, net‐erosion of particulate organic C might export another important fraction of the NEP from terrestrial ecosystems to aquatic systems, further decreasing the C sink actually stored on terrestrial ecosystems.

**TABLE 2:** Global and regional C and dissolved organic carbon (DOC) ratios. DOC, net primary production (NPP) and net ecosystem productivity (NEP) and export ratios

	DOC leaching (pg C year^−1^)	NPP (pg C year^−1^)	NEP (pg C year^−1^)	DOC_LCH_/NEP (%)
Boreal	0.02	7.61	0.23	10.44
Temperate	0.05	17.03	0.47	10.51
Tropic	0.16	45.24	0.74	22.23
Subtropic	0.05	18.05	0.45	10.08
Global	0.28	87.93	1.92	14.69

### 3.4 Historical changes in DOC leaching flux

**FIGURE 9:**
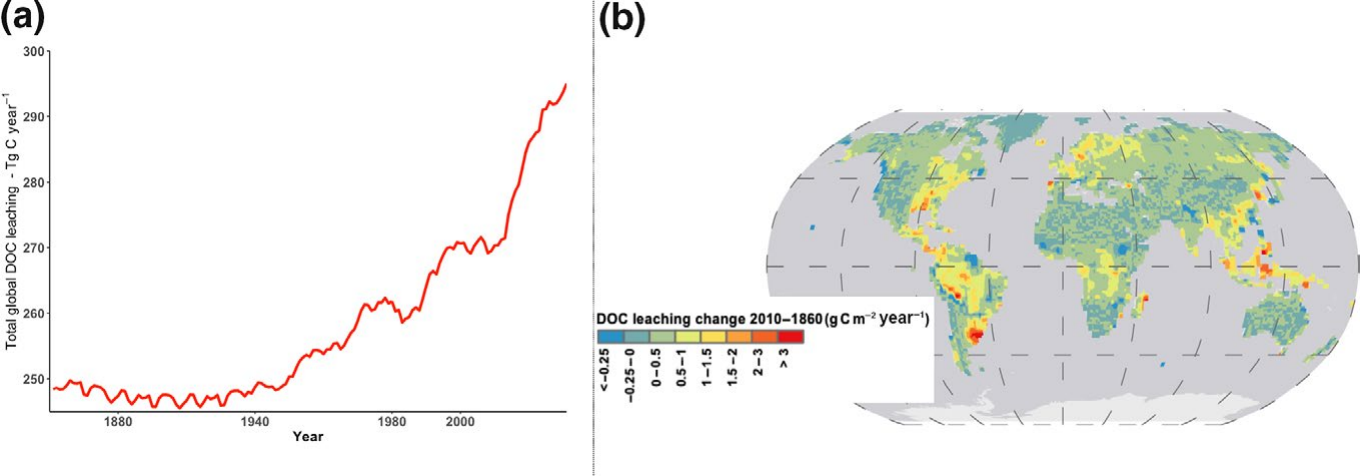
Global dissolved organic carbon leaching flux (a) temporal and (b) spatial changes

For the period 1861–1870, we estimate an average global terrestrial DOC leaching of 250 Tg C year^−1^, which then increases by 35 Tg C year^−1^ (14%; Figure 9b). Detailing these estimates per major climate zones, we simulated the highest relative increase of 28% in the boreal zone, followed by an increase of 22% in the temperate and 18% in the subtropical zones, and the lowest increase of 9% in the tropical zone (Figure 9).

Our results show a concomitant increase in global runoff over the historical period of 15.5% (Table S5; Figure S9a) with a significant temporal and spatial correlation between runoff and DOC leaching (*R* ^2^ = 0.75 and 0.49 respectively), which can be explained by a strong transport limitation for DOC leaching flux from soils. However, the highest relative increase in DOC leaching in the Boreal zone is primarily due to the fact that here the highest increase in runoff is simulated, in line with a recent study in Sweden (Nydahl et. al., 2017). In contrast, the temperate, subtropical and tropical zones show an increase in DOC leaching which appears to be mainly driven by an increase in NPP (Table S4; Figure S9b) and only in the second place by an increase in runoff.

Over the simulation period, surface soil leaching contributes about 90% to total DOC leaching from soil. However, the surface soil layer contains only ~40% of total soil DOC stocks.

### 3.5 Sensitivity analysis

A sensitivity analysis was performed to constrain the uncertainties in DOC stocks and fluxes reported in the previous sections. To do so, an uncertainty analysis on estimated DOC stocks was performed, by repeating the simulation with the observation‐based WATCH meteorological forcing data (Weedon et al., 2010), instead of CRU‐NCEP (Figure S10). Furthermore, we ran the model with the CRU‐NCEP configuration and the second‐best combination of *K*_p_ and *K*_DOC_ with regard to RMSE (Figure S11) as well as a parameter set using as *K*_p_ and *K*_DOC_ for the PFTs that were not calibrated, the recalibrated *K*_p_ and *K*_DOC_ values from the PFTs that were most similar to them (as opposed to keeping the default parameters for non‐calibrated PFTs; Figure S12).

Using the WATCH forcing, simulated surface soil and total soil DOC stocks at global scale are 0.11 and 0.22 Pg C (21% and 35% difference from the main run) respectively. Using CRU‐NCEP with the second‐best parameter set or with the recalibrated parameters to all PFTs, the surface soil DOC stock is estimated at 0.17 and 0.15 Pg C, respectively (21% and 7% difference from the baseline run), and the total DOC stock is estimated at 0.43 and 0.37 Pg C respectively (27% and 9% difference from the baseline run). Average DOC concentration is 30.89 and 29.04 mg C L^−1^ in the surface soil (5% and 1% difference from the baseline run) and 10.19 and 8.60 mg C L^−1^ in the sub‐surface soil (23% and 4% difference from the baseline run) for the second‐best parameter set and the application of recalibrated parameters to all PFTs respectively. These differences between simulations indicate the need for more soil DOC measurements covering the broad range of climate and vegetation types to narrow down estimates. However, as the magnitude of these differences is not extremely large, our confidence in the order of magnitude of the final results is high.

## 4 MODEL LIMITATIONS AND FUTURE DIRECTIONS

As mentioned earlier, in JULES‐DOCM, peatlands and organic soil representations are still missing due to model limitations. Peatlands cover a small part of the total land area and they are an important terrestrial C storage (Blodau, 2002; Leifeld & Menichetti, 2018), with high DOC concentrations in the soil solution (Billett et al., 2010). Hence, future model development should be focused on the representation of peatlands and organic soils and their contribution to soil DOC dynamics.

In JULES‐DOCM, we implemented a soil module which includes the main controls on the DOC dynamics, which are temperature, precipitation and vegetation type, but still lacks the representation of other environmental drivers such as pH and nutrient levels, which have been suggested to have a controlling role in soil DOC dynamics (Kalbitz et al., 2000). For instance, plant and soil organic matter C:N ratios can significantly influence DOC degradability and, therefore, its leaching (Sanderman et al., 2009; Van den berg et al., 2012). Thus, including these drivers could improve the modelling of soil DOC processes, including their temporal and spatial dynamics.

A larger database of DOC observations, with better spatial coverage and more simultaneous measurements of DOC and SOC, would be key to improving model parameterizations. In addition, data are not evenly geography distributed, most of them originating from parts of Europe and the United States with limited data coverage from the tropical biome (Hartmann et al., 2014). Hence, collecting a database that better covers the different biomes at the global scale will help refining the model parameterization. In particular, as our results show a significant contribution of the tropical zone to soil DOC and DOC leaching flux, more data from tropical zone will help better representation of the tropical biome. In addition, soil moisture and runoff are important controllers of the soil DOC concentration and leaching. The hydrology module has been carefully evaluated and applied globally by Gedney and Cox (2003) and Gedney et al. (2006). Nevertheless, further improvements to this module will be instrumental for the quantitative assessment of soil DOC stocks and leaching fluxes by JULES‐DOCM. In this context, more measurements of DOC concentration in headwater streams will also help to improve the calibration of DOC leaching fluxes in the future.

Our study shows that DOC leaching represents a significant fraction (approximately 15%) of NEP globally. Therefore, national or regional land C budgets relevant to the Nationally Determined Contributions (NDCs) that are at the heart of the Paris Agreement need to account for the C exported from land ecosystems to the rivers to ocean continuum. Similarly, dynamic global vegetation models and their associated ESMs also need to include all forms of C exports from land, and their fate through the river network to the oceans, to avoid overestimating the terrestrial C sink. With our improved version of JULES‐DOCM, we will be able to study the future trend for the DOC leaching flux from soil to river system at the global scale and CO_2_ fertilization, climate and land use change impact on it. We believe that our work highlights the necessity for including lateral C fluxes in global C budgeting.

## CONFLICT OF INTEREST

The authors declare no conflict of interest.

## Supporting information

Supplementary Material

## Data Availability

All simulation results and source code of the updated, globally calibrated version of JULES‐DOCM can be found at https://code.metoffice.gov.uk/svn/jules/main/branches/dev/mahdinakhavali/vn4.4_JULES_DOCM_GLOBAL_NAKHAVALI/ (registration is required).
