# Supplementary material for: Leaching of dissolved organic carbon from mineral soils plays a significant role in the terrestrial carbon balance
Source: Glob Chang Biol. 2020 Dec 14;27(5):1083–96. doi: 10.1111/gcb.15460 (PMC7898291; doi:10.1111/gcb.15460)
Supplement: Supplementary file 1 — Supplementary Material [file GCB-27-1083-s001.pdf]

Supporting document:

Title

Leaching of dissolved organic carbon from mineral soils plays a significant role in the terrestrial carbon balance

Authors

Mahdi Nakhavali[1,2]*, Ronny Lauerwald[2], Pierre Regnier[2], Bertrand Guenet[3], Sarah Chadburn[1] and Pierre Friedlingstein[1,4]

Affiliations

[1]University of Exeter, College of Engineering, Mathematics and Physical Sciences, Exeter, EX4 4QE, UK

[2]Biogeochemistry and Modelling of the Earth System, Department Geoscience, Environment and Society, Université Libre de Bruxelles, Bruxelles, Belgium

[3]Laboratoire de Géologie de l'ENS, PSL Research University, Paris, France

[4]Laboratoire de Meteorologie Dynamique, Institut Pierre-Simon Laplace, CNRS-ENS-UPMC-X, Departement de Geosciences, Ecole Normale Superieure, 24 rue Lhomond, 75005 Paris, France

TABLE S1-6

FIGURES S1-12

Table S1. Dissolved Organic Carbon measurements from literature and database

SITE	DOC in soil- Annual mean (mg C L^{-1})	Depth of measurement (cm/horizon)	Reference
**Boreal Forest**			
Coulissenhieb	24.7	20	1
Coulissenhieb	5.0	90	1
Bikenes	50.6	9	2
Waldstein	47.8	12	2
Kangasvaara	38.5	E horizon*	3
Kangasvaara	0.5	B horizon	3
**Temperate forest**			
Nagano	3.23	O,A horizons	4
Tango	20.95	O,A horizons	4
Tango	2.3	B horizon	4
Kyoto	14.7	O,A horizons	4
Kyoto	4.3	B horizon	4
Oregon	40	10	5
Oregon	25.5	20	5
Oregon	15.75	30	5
Oregon	0.5	70	5
South Carolina	19.22	15	6
South Carolina	1.39	50	6
South Carolina	0.97	175	6
South Carolina	0.82	600	6
Steigerwald	17.8	20	7
Steigerwald	5.7	60	7
Søro	22.9	15	8
Søro	14.1	100	8
Laois	10.98	15	8
Laois	3.2	70	8
Loobos	32.1	5	8
Loobos	5.6	120	8
Wetzsein	43.1	20	8
Wetzsein	17.6	90	8
Hainich	7.5	10,20	9
Brasschaat	34	10,35	10
Brasschaat	22	75	10
Ebrach	23.1	20	11
Ebrach	9.9	120	11
Freising	6.6	30	11
Freising	3.0	140	11
Mitterfels	1.3	30	11

Mitterfels	1.2	120	11
Ebersberg	7.5	30	11
Ebersberg	3.6	100	11
Flossenbürg	3.3	20	11
Flossenbürg	4.3	120	11
Goldkronach	4.9	20	11
Goldkronach	3.0	120	11
Kreuth	5.2	20	11
Kreuth	2.4	120	11
Rothenkirchen	5.5	30	11
Rothenkirchen	2.9	80	11
Sonthofen	5.8	20	11
Sonthofen	4.7	120	11
Zusmarshausen	4.5	30	11
Zusmarshausen	2.4	130	11
Landau	13.0	30	11
Landau	6.7	130	11
Riedenburg	5.4	30	11
Riedenburg	5.0	120	11
Würzburg	3.0	30	11
Würzburg	7.7	110	11
Altdorf	7.3	140	11
Dinkelsbühl	6.2	30	11
Dinkelsbühl	7.2	120	11
Pegnitz	15.4	20	11
Pegnitz	6.4	120	11
**TROPICAL FOREST**			
Bukit Soeharto	25.95	O,A horizons	12
Bukit Soeharto	9.9	B1 horizon	12
Bukit Bankirai	21.85	O,A horizons	12
Bukit Bankirai	6	B1 horizon	12
La Selva	3.9	25	13
La Selva	0.7	150	13
Kuaro_KR1	13.4	A1	14
Kuaro_KR1	5.4	B1	14
Kuaro_KR2	5.7	A	14
Kuaro_KR2	1.7	Bt1	14
Kuaro_KR3	10.1	A	14
Kuaro_KR3	2.8	Bt1	14
Campinarana	50.4	10,20	15
Bacia3	7.38	10,20	15
Thurston	3	20 to 35	16

Ola'a	6	20 to 35	16
Laupahoehoe	2.1	20 to 35	16
Kohala	1.8	20 to 35	16
Kolekole	3.3	20 to 35	16
Kokee	4.3	20 to 35	16
Korhogo	3.20		17
Bouake	1.85		17
Divo	9.65		17
Azaguie, Angededou,Adiopodoume	10.77		17
Juruena, Mato Grosso,	17.95		18
Juruena, Mato Grosso	4.92		18
Tanguro Ranch, Mato Grosso	31.8		19
Tanguro Ranch, Mato Grosso	14.4		19
Fazenda Victoria	17.8		20
Fazenda Victoria	6.12		20
**SUBTROPICAL FOREST**			
Dinghushan	22.66	0 to 20	21
Guandaushi	12.5	15	22
Guandaushi	10.3	30	22
Gao County, Sichuan Provincem China	98.82		23
**Grassland**			
Carlow	13	10 to 28	24
Carlow	5	28 to 79	24
Easter Bush	17.1	30	8
Easter Bush	16.3	100	8
Früebüel	3.7	30	8
Früebüel	12.3	100	8
Laqueuille	2.04	30	8
Laqueuille	1.7	90	8
**Cropland**			
Carlow	4.2	0 to 40	24
Klingenberg	13.9	35	8
Klingenberg	10.1	75	8
Grignon	17.3	0 to 40	8
Grignon	13.8	90	8

1. Michalzik et al. (1999)(Michalzik, Michalzik, Matzner, & Matzner, 1999)(Michalzik, Michalzik, Matzner, & Matzner, 1999)(Michalzik, Michalzik, Matzner, & Matzner, 1999)(Michalzik, Michalzik, Matzner, & Matzner, 1999)(Michalzik, Michalzik, Matzner, & Matzner, 1999)(Michalzik, Michalzik, Matzner, & Matzner, 1999)(Michalzik, Michalzik, Matzner, & Matzner, 1999) 2.Mulder et al. (2000) 3.Piirainen et al. (2004) 4.Fujii, Funakawa, et al. (2011) 5. Yano et al. (2004) 6. Markewitz & Richter (1998) 7.Solinger et al.(2001) 8. Kindler & Siemens (2010) 9. Kutsch et al. (2010) 10. Gielen et al. (2011) 11.Borken et al. (2011) 12.Fujii et al. (2009) 13. Schwendenmann & Veldkamp (2005) 14.Fujii, Hartono, et al. (2011) 15.Cornu *et al.* (1997) 16.Hedin et al (2003) 17.Roose and Lelong (1981) 18.Johnson et al. (2006) 19. Neu *et al.* (2016) 20. Markewitz *et al.* (2004) 21.Fang et al. (2009) 22.Liu & Sheu (2003) 23.Justine *et al.* (2017) 24.Walmsley et al. (2011)

* Horizons O, A and E are treated as surface soil (down to 35 cm) and Horizon B is treated as sub-surface soil (from 35 cm down to 3 m)

Note however, the observed data for boreal zone only covers needle-leaf evergreen trees (NET) which cover a large proportion of the boreal biome. Needle-leaf deciduous forest (NDT) which covers another important portion of boreal biome is not covered by our literature database. Hence, we used the default DOC residence time and production for NDT, which is about 1/5 of the NET residence time.

Table S2. DOC stock, production and decomposition, as well as ratios of DOC leaching to DOC production, DOC leaching to DOC decomposition and DOC leaching to terrestrial NPP for the 4 biomes

	DOC stock (Tg C)	DOC production (Tg C yr-1)	DOC decomposition (Tg C yr-1)	DOC production (g C m^{-2} yr^{-1})	DOC decomposition (g C m^{-2} yr^{-1})	DOC leaching divided		NPP (Tg C yr-1)
						production	decomposition	
Boreal	70	104	39	3.13	1.17	23.07	61.51	0.31
Temperate	97	244	96	8.46	3.29	20.5	52.03	0.28
Tropic	101	858	341	24.77	9.85	19.34	48.68	0.37
Sub-tropic	70	273	113	4.32	1.77	16.48	39.82	0.26

Table S3. Simulation results by using WATCH and CRU_NCEP with default, second best model parameter set and parameter set for all PFTs

WATCH
Surface/total stock (Pg C $year^{-1}$) = 0.108 ; 0.22
Surface/Sub-surface concentration (mg C L^{-1}) = 28.64 ; 3.1

CRU_NCEP default parameter set
Surface/total stock (Pg C $year^{-1}$) = 0.142 ; 0.34
Surface/Sub-surface concentration (mg C L^{-1}) = 26.03 ; 7.62

CRU_NCEP with 2nd best guess parameter set
Surface/total stock (Pg C $year^{-1}$) = 0.170 ; 0.43
Surface/Sub-surface concentration (mg C L^{-1}) = 30.89 ; 10.19

CRU_NCEP with parameter set for all PFTs
Surface/total stock (Pg C $year^{-1}$) = 0.154 ; 0.37
Surface/Sub-surface concentration (mg C L^{-1}) = 29.04 ; 8.60

Table S4. Multiple linear regression of total DOC vs environmental controllers

multiple linear regression	
	r2
Total DOC stock vs smcl*+npp+soc	0.6174667
Total DOC stock vs npp+soc	0.6170734
Total DOC stock vs smcl+soc	0.596213
Total DOC stock vs smcl+npp	0.3429866

* smcl = soil moisture

Table S5. Historical and present day averaged runoff

	Global	Boreal	Temperate	Tropics	Sub-tropics
**NPP (avg. 1860s)**(Pg C yr^{-1})	71	6	13	38	14
**NPP (avg. 2000s)**(Pg C yr^{-1})	92	8	18	47	19
**Runoff (avg. 1860s)** (Kg water m^{-2} yr^{-1})	31528	2708	4883	20029	3908
**Runoff (avg. 2000s)** (Kg water m^{-2} yr^{-1})	36409	3539	5718	22658	4494

Table S6. Model evaluation at selected tropical sites for which observational data are available

	Rainfall (mm)		Temperature (•C)		SOC (kg C m-2)		Soil respiration (kg C m-2)		Runoff mm		Soil DOC (mg C L-1) Surface soil		Soil DOC (mg C L-1) Subsurface soil	
SITE	mes.	mod.	mes.	mod.	mes.	mod.	mes.	mod.	mes.	mod.	mes.	mod.	mes.	mod.
Tanguro Ranch, Mato Grosso, Brazil[*11]	1905	1832.76	25	26.6	7.2	8.08	-	1.53	-	636.43	31.8	34.52	14.4	8.68
Fazenda Victoria[*12]	1803	2380.82		27.2	11.8	7.65	1.88	1.63	-	1244.01	17.8	29.25	6.12	7.60
near Juruena, Mato Grosso, Brazil[*10]	2200	2128.53	24	25.83	7.5	8.02	-	1.63	-	924.52	17.95	31	4.92	7.89
La Selva[*7]	4073	3713.62	24.6	25.58	7.1[*2]	8	1.27 [*1]	1.87	-	1948.6	3.9	14	0.7	2.8
Bukit Soeharto, Bukit Bankirai, Kuaro (KR1,2,3) [*6]	2307	2391.67	27	27.18	3.9	6.9	0.71	1.82	1301	1007.03	11.4	9	-	-
Campinarana, Bacia 3[*8]	2100	2522.05	26	26.68	-	5.36	-	1.57	-	1351.58	28.89	26	-	-
Ivory Coast (Korhogo)[*9]	1350	1103.68	27	27.26	4.2	5.08	-	0.71	289	117.69	-	-	3.20	6.89
Ivory Coast (Bouake)[*9]	1200	1254.41	26.1	26.7	6.9	6.17	-	0.88	174	148.52	-	-	1.85	6.66
Ivory Coast (Divo)[*9]	1550	1254.41	26	26.7	6.9	6.17	-	0.88	250	148.52	-	-	9.65	7.16
Ivory Coast (Azaguie, Angededou, Adiopodoume) [*9]	1750	1379.1	26.2	26.99	11.7	6.49	-	0.98	462	155.52	-	-	10.77	7.56

[*1](Schwendenmann et al., 2003),[*2](Veldkamp et al., 2003),[*3](E. Roose, 1981),[*4](Bonini et al., 2018),[*5](Novaes et al., 2009), [*6](Fujii et al. 2009), [*7](Schwendenmann & Veldkamp 2005), [*8](Cornu *et al.* 1997), [*9](Roose and Lelong 1981), [*10](Johnson et al. 2006) [*11] (Neu *et al.* 2016) [*12] Markewitz *et al.* (2004)

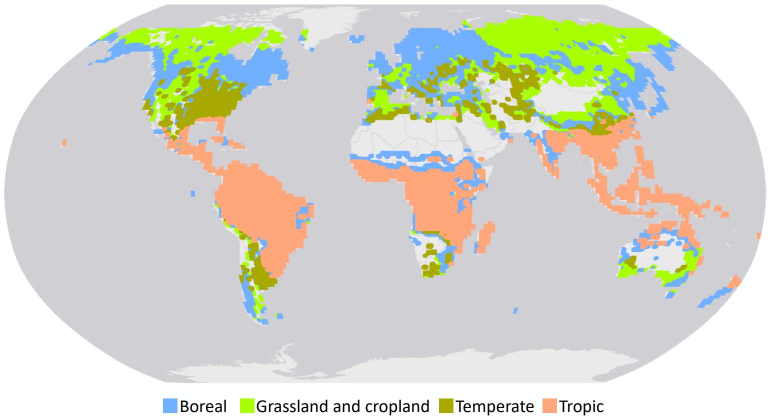

Figure S1. Ecosystem type categories in JULES-DOCM

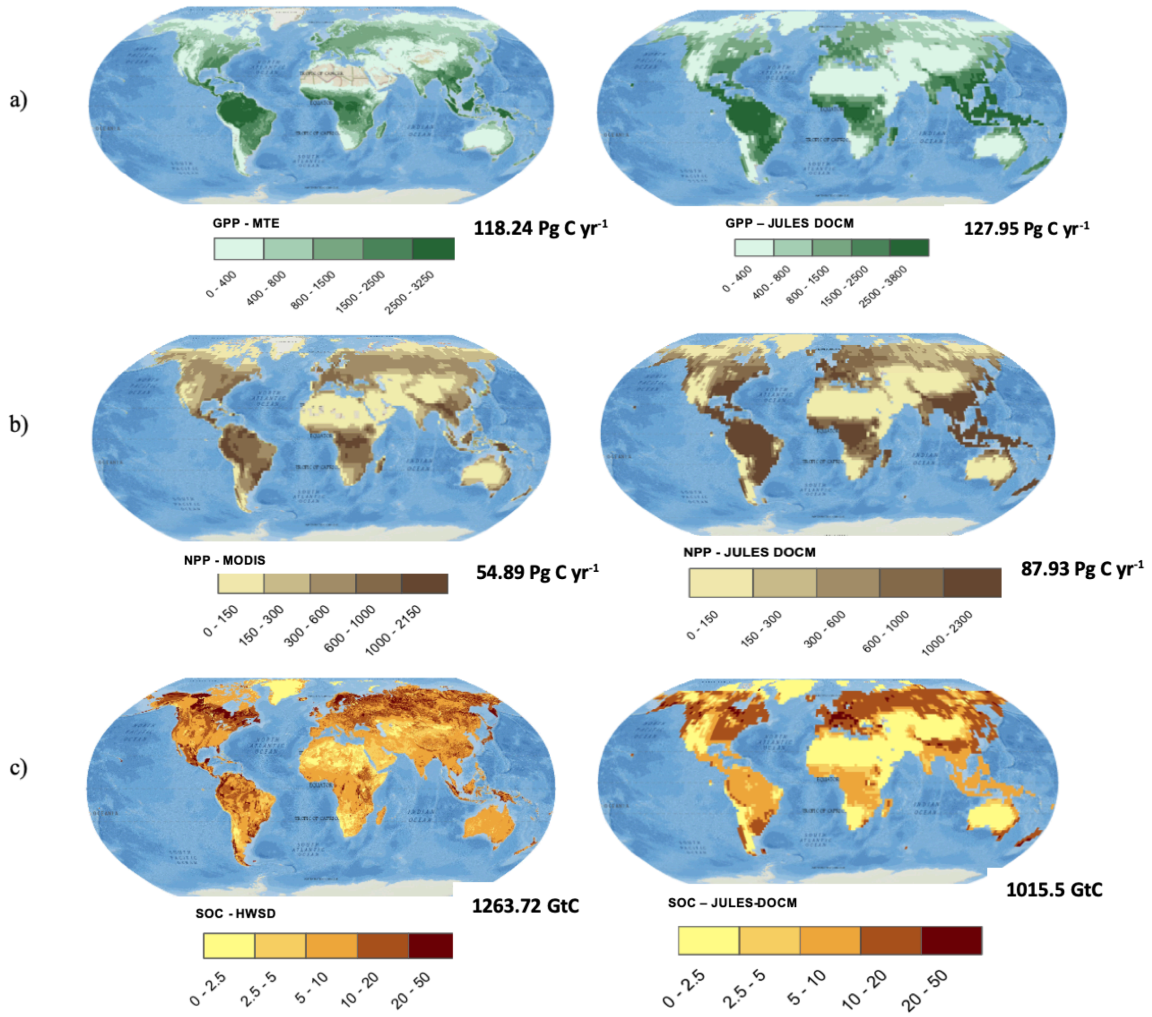

Figure S2. Comparison of a) gross primary production (GPP) from model tree ensemble (MTE) (Jung et al. 2009) vs JULES-DOCM, b) net primary production (NPP) from MODIS 17 (Zhao, Heinsch, Nemani, & Running, 2005; Zhao & Running, 2010) vs JULES-DOCM and c) surface and sub-surface soil layer SOC from Harmonized World Soil Database (HWSD) (Nachtergaele et al., 2010) vs JULES-DOCM. Globally integrated values are also reported.

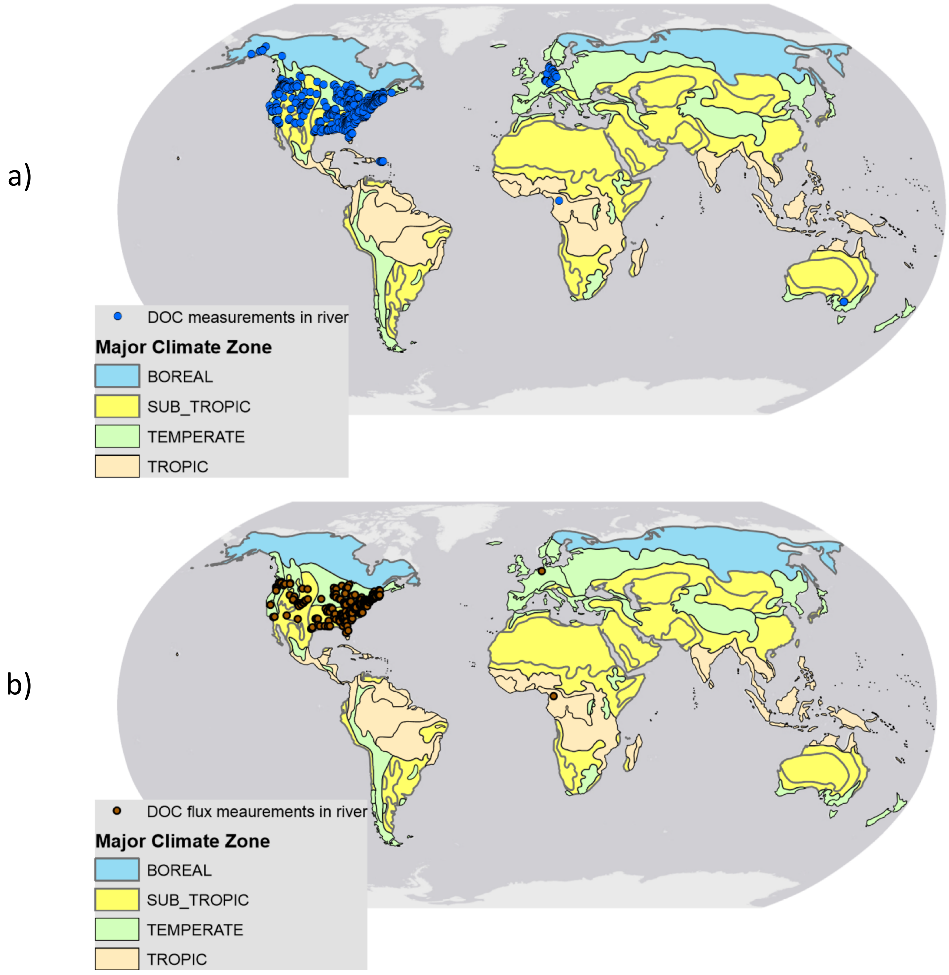

Figure S3. Locations of measurements of dissolved organic carbon a) concentration in runoff in mg C L-1 b) leaching flux (calculated from measured DOC concentration and instantaneous discharge) in g C m-2 yr-1, using the COSCAT scheme for regionalisation per major climate zones. Blue and brown points indicate measurements from the GloRiCh database (Hartmann, Lauerwald, & Moosdorf, 2014). Black lines with blue area indicates COSCAT (costal segmentation and related catchments, (Meybeck, Dürr, & Vörösmarty, 2006)

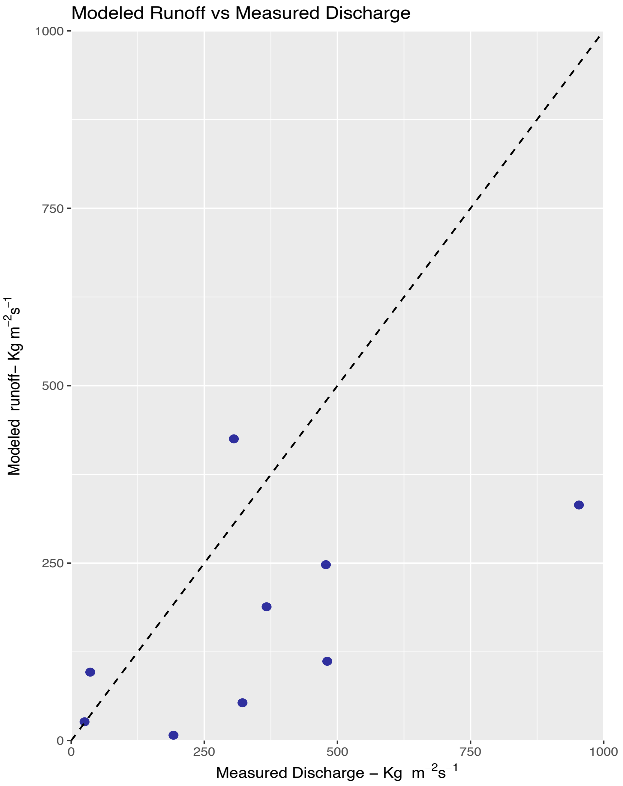

Figure S4. Comparison of modelled runoff against measured discharge from GloRiCh data at COSCAT zones where the DOC concentration measurements were compared to modelled (units in Kg m^{-2} s^{-1})

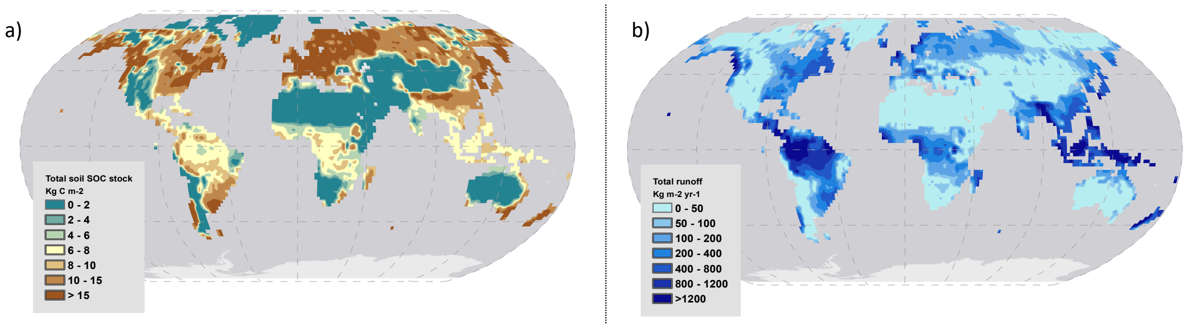

Figure S5. Simulated present-day a) soil SOC stock (kg C m^{-2}) and b) runoff (kg m^{-2} y^{-1})

We studied environmental factors that could have an impact on the spatial variability of DOC stocks in surface soil. From all the studied factors, soil moisture, NPP and SOC stocks had the highest correlation with surface soil DOC stock (r^2 of 0.24, 0.42 and 0.48 respectively). Although runoff had a high correlation with the DOC leaching flux, it did not show a significant correlation to DOC stocks. A multiple linear regression analysis confirmed that SOC is the main control of the spatial variations in soil DOC stocks (Table S4), NPP and soil moisture only adding little explanatory power. We thus conclude that the correlation between NPP or soil moisture and DOC is mainly due to the correlation of those variables with SOC.

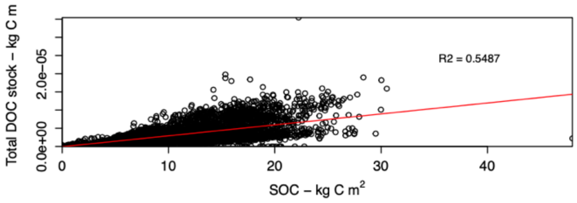

Figure S6. Total soil DOC stock vs SOC for individual grid cells

However, closer inspection reveals that all regions of high DOC stocks correspond to regions of high SOC stocks. The most important are Western Europe and the Eastern part of North America, significant portions of South-East Asia, and for the Southern Hemisphere, New Zeeland, Eastern Australia and a portion of South America under subtropical to temperate climate. However, the converse is not true, and large portions of the globe with high to very high SOC stocks have low DOC stocks. This feature is most prominent in the broad boreal region (most of Canada and Russia), but is also observable in arid parts of temperate to subtropical North America and Asia.

In our soil DOC model, the DOC stock is equal to the product of the SOC stock times the dimensionless ratio of the time constants $K_{prod}/(K_{leach}+K_{dec})$. This dimensionless ratio (Figure S7) is comprised between 0.06 and 1.5 per grid-cell and is lowest in the boreal region (0.36 on average) while the tropics and subtropics overall show highest values (0.68 and 0.59, respectively). Although there are some local exceptions, the temperate region is characterized by intermediate values (0.45). Therefore, the latitudinal pattern in K_{prod} can also be observed in the dimensionless ratio $K_{prod}/(K_{leach}+K_{dec})$, but the variation is now significantly smaller (about a factor of 2). This result can be explained by the concomitant increase in K_{dec} and K_{leach} from high to low latitudes, the time constants scaling to temperature and runoff.

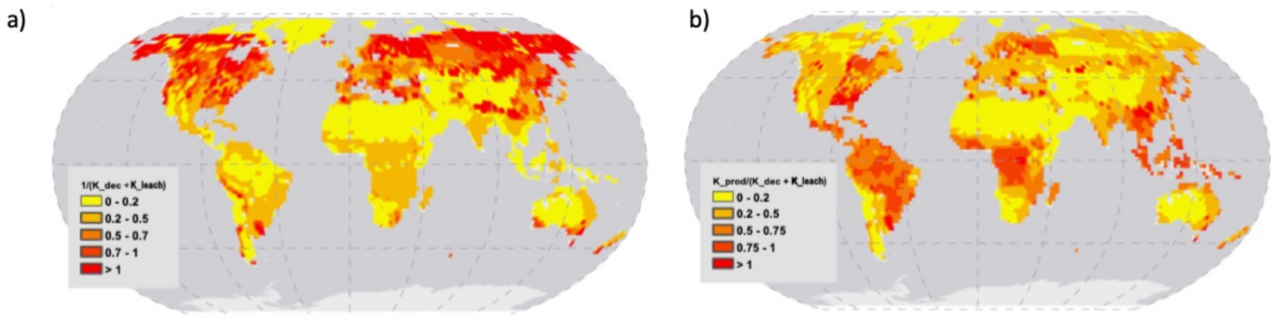

Figure S7. a) Loss term and b) production over loss terms

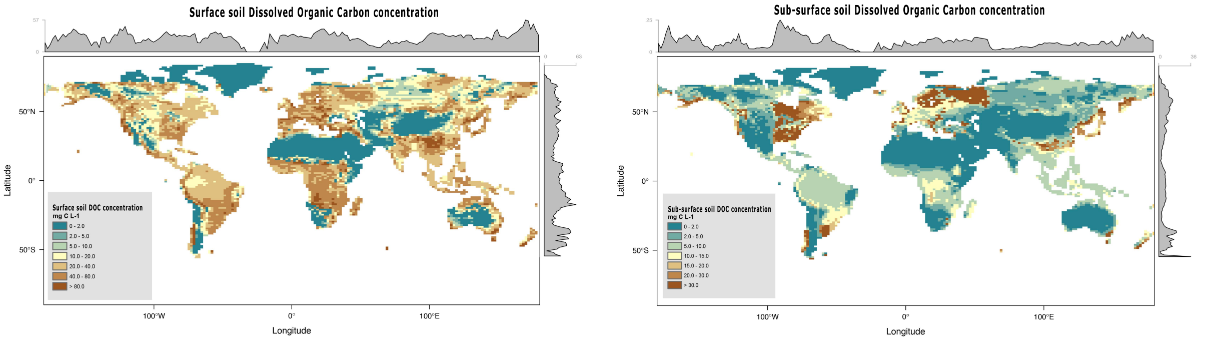

Figure S8. DOC concentration in the surface and subsurface soils.

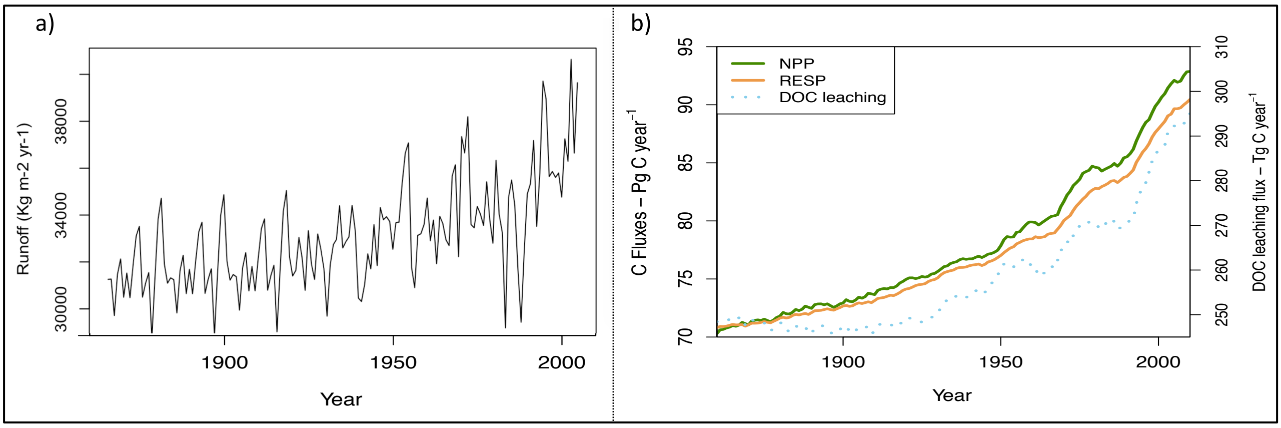

Figure S9. historical changes of a) total runoff and b) NPP, soil respiration (RESP) and DOC leaching flux

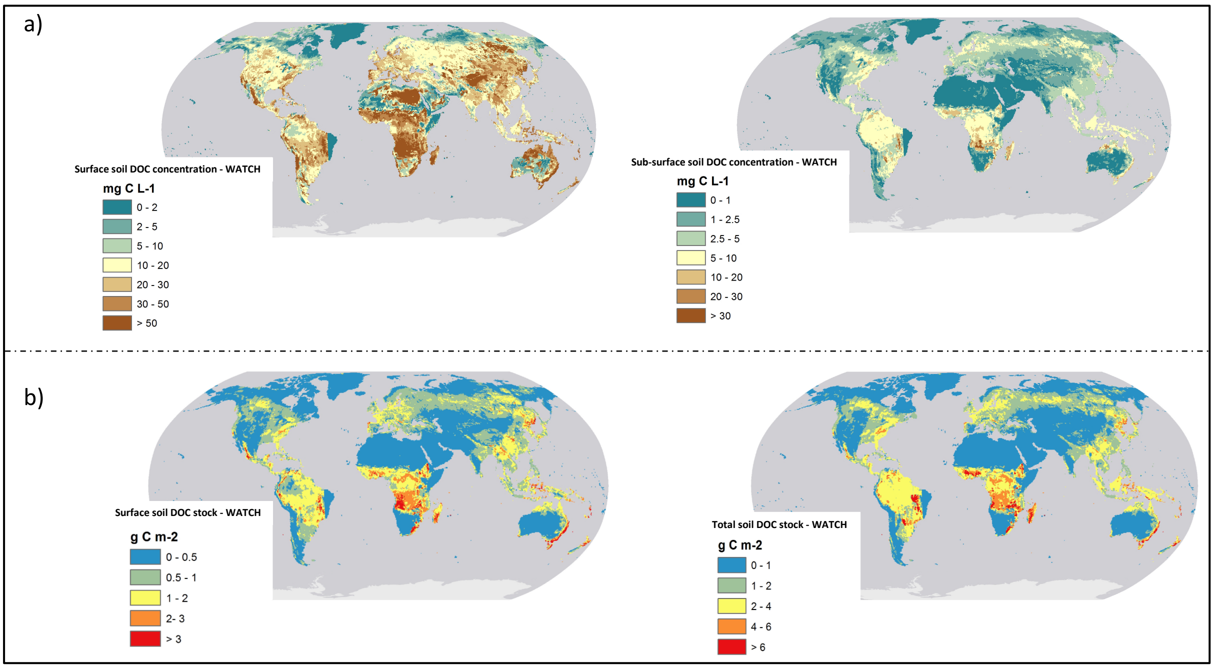

Figure S10. a) DOC concentration at the surface and subsurface soils b) DOC stocks at surface and total soils using WATCH data

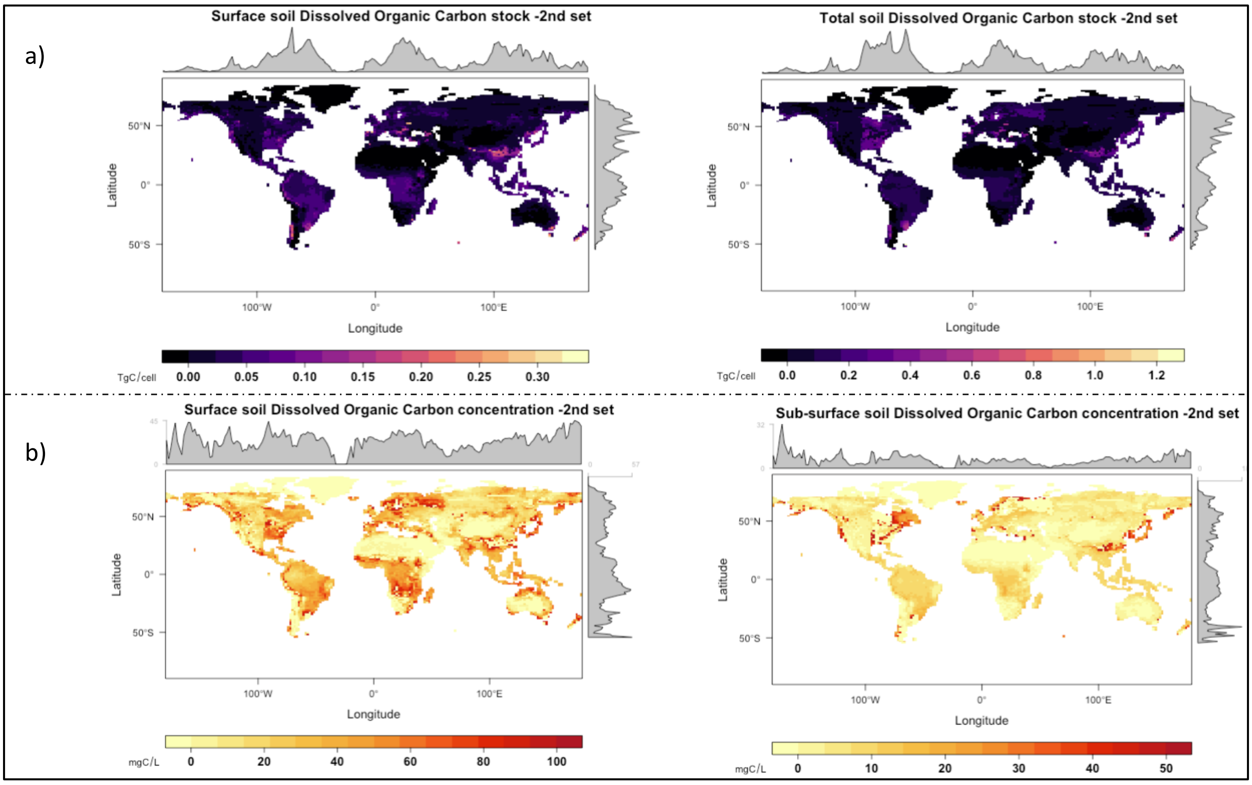

Figure S11. a) DOC concentration at the surface and subsurface soils b) DOC stocks at surface and total soils using second-best combination of K_P and K_{DOC}

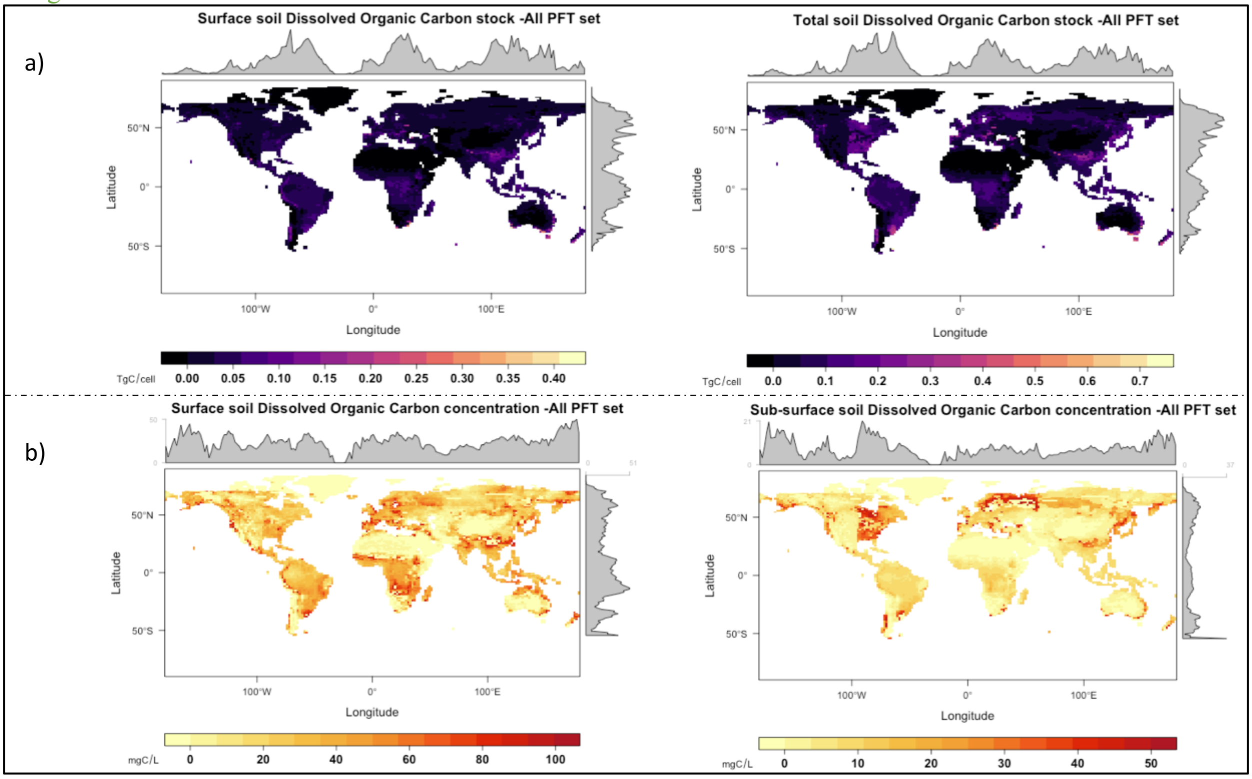

Figure S12. a) DOC concentration at the surface and subsurface soils b) DOC stocks at surface and total soils using as K_P and K_{DOC} for the PFTs that were not calibrated, the recalibrated K_P and K_{DOC} values from the PFTs that were most similar to them

Reference

Bonini, I., Hur Marimon-Junior, B., Matricardi, E., Phillips, O., Petter, F., Oliveira, B., & Marimon, B. S. (2018). Collapse of ecosystem carbon stocks due to forest conversion to soybean plantations at the Amazon-Cerrado transition. *Forest Ecology and Management*, *414*(January), 64–73. https://doi.org/10.1016/j.foreco.2018.01.038

Borken, W., Ahrens, B., Schulz, C., & Zimmermann, L. (2011). Site-to-site variability and temporal trends of DOC concentrations and fluxes in temperate forest soils. *Global Change Biology*, *17*(7), 2428–2443. https://doi.org/10.1111/j.1365-2486.2011.02390.x

Cornu, S., Ambrosi, J. ., Luca, Y., & Fevrier, D. (1997). A comparative study of the soil solution chemistry of two Amazonian forest soils (Central Amazonia, Brazil). *Hydrology and Earth System Sciences*, *1*(2), 313–324.

Fang, Y., Zhu, W., Gundersen, P., Mo, J., Zhou, G., & Yoh, M. (2009). Large loss of dissolved organic nitrogen from nitrogen-saturated forests in subtropical China. *Ecosystems*, *12*(1), 33–45. https://doi.org/10.1007/s10021-008-9203-7

Fujii, K., Funakawa, S., Shinjo, H., Hayakawa, C., Mori, K., & Kosaki, T. (2011). Fluxes of dissolved organic carbon and nitrogen throughout Andisol, Spodosol and Inceptisol profiles under forest in Japan. *Soil Science and Plant Nutrition*, *57*(6), 855–866. https://doi.org/10.1080/00380768.2011.637304

Fujii, K., Hartono, A., Funakawa, S., Uemura, M., & Kosaki, T. (2011). Fluxes of dissolved organic carbon in three tropical secondary forests developed on serpentine and mudstone. *Geoderma*, *163*(1–2), 119–126. https://doi.org/10.1016/j.geoderma.2011.04.012

Fujii, K., Uemura, M., Hayakawa, C., Funakawa, S., Sukartiningsih, Kosaki, T., & Ohta, S. (2009). Fluxes of dissolved organic carbon in two tropical forest ecosystems of East Kalimantan, Indonesia. *Geoderma*, *152*(1–2), 127–136. https://doi.org/10.1016/j.geoderma.2009.05.028

Gielen, B., Neirynck, J., Luyssaert, S., & Janssens, I. A. (2011). The importance of dissolved organic carbon fluxes for the carbon balance of a temperate Scots pine forest. *Agricultural and Forest Meteorology*, *151*(3), 270–278. https://doi.org/10.1016/j.agrformet.2010.10.012

Hartmann, J., Lauerwald, R., & Moosdorf, N. (2014). A Brief Overview of the GLObal RIver Chemistry Database, GLORICH. *Procedia Earth and Planetary Science*, *10*, 23–27. https://doi.org/10.1016/j.proeps.2014.08.005

Hedin, L. O., Vitousek, P. M., & Matson, P. A. (2003). Nutrient losses over four million years of tropical forest development. *Ecological Society of America*, *84*(9), 2231–2255.

Johnson, C. E., Driscoll, C. T., Siccama, T. G., & Likens, G. E. (2000). Position and Landscape in a Northern Hardwood Watershed Ecosystem. *Gene*, *3*(2), 159–184.

Johnson, M., Lehmann, J., Selva, E. C., Abdo, M., Riha, S., & Guimar, E. (2006). Organic carbon fluxes within and streamwater exports from headwater catchments in the southern Amazon. *Hydrological Processes*, *20*, 2599–2614. https://doi.org/10.1002/hyp.6218

Justine, M. F., Yang, W., Wu, F., Tan, B., Khan, M. N., & Li, Z. (2017). Dissolved organic matter in soils varies across a chronosequence of Pinus massoniana plantations. *Ecosphere*, *8*(April), 1–11. https://doi.org/10.1002/ecs2.1764

Kindler, R., & Siemens, J. (2010). Dissolved carbon leaching from soil is a crucial component of the net ecosystem carbon balance. *Global Change …*, 1167–1185. https://doi.org/10.1111/j.1365-2486.2010.02282.x

Kutsch, W. L., Persson, T., Schrumpf, M., Moyano, F. E., Mund, M., Andersson, S., & Schulze, E.-D. (2010). Heterotrophic soil respiration and soil carbon dynamics in the deciduous Hainich forest obtained by three approaches. *Biogeochemistry*, *100*(1–3), 167–183. https://doi.org/10.1007/s10533-010-9414-9

Liu, C. P., & Sheu, B. H. (2003). Dissolved organic carbon in precipitation, throughfall, stemflow, soil solution, and stream water at the Guandaushi subtropical forest in Taiwan. *Forest Ecology and Management*, *172*(2–3), 315–325. https://doi.org/10.1016/S0378-1127(01)00793-9

Markewitz, D., Davidson, E., Moutinho, P., & Nepstad, D. (2004). NUTRIENT LOSS AND REDISTRIBUTION AFTER FOREST CLEARING ON A HIGHLY WEATHERED SOIL IN AMAZONIA. *Ecological Applications*, *14*(August 2002), 177–199.

Markewitz, D., & Richter, D. D. (1998). The bio in Aluminum and Silicon Geochemistry. *Biogeochemistry*. https://doi.org/10.1023/A

Meybeck, M., Dürr, H. H., & Vörösmarty, C. J. (2006). Global coastal segmentation and its river catchment contributors: A new look at land-ocean linkage. *Global Biogeochemical Cycles*, *20*(1), 1–15. https://doi.org/10.1029/2005GB002540

Michalzik, B., Michalzik, B., Matzner, E., & Matzner, E. (1999). Dynamics of dissolved organic nitrogen and carbon in a Central European Norway spruce …. *European Journal of Soil Science*, (December), 579–590. https://doi.org/10.1046/j.1365-2389.1999.00267.x

Moore, T. R., & Clarkson, B. R. (2007). Dissolved organic carbon in New Zealand peatlands. *New Zealand Journal of Marine and Freshwater Research*, *41*(1), 137–141. https://doi.org/10.1080/00288330709509902

Mulder, J., Gallardo, L., JF, M., E., T., E., B., C., C., … EstebanA., V. (2000). *Effects of natural climatic variations on production and transport of dissolved organic matter in European forest ecosystems*.

Nachtergaele, F., Velthuizen, H. van, Verelst, L., Batjes, N. H., Dijkshoorn, K., Engelen, V. W. P. van, … Montanarela, L. (2010). The Harmonized World Soil Database. *Proceedings of the 19th World Congress of Soil Science, Soil Solutions for a Changing World, Brisbane, Australia, 1-6 August 2010*, 34–37. https://doi.org/3123

Neu, V., Ward, N. D., Krusche, A. V, & Neill, C. (2016). Dissolved Organic and Inorganic Carbon Flow Paths in an Amazonian Transitional Forest. *Frontiers in Marine Science*, *3*(June), 1–15. https://doi.org/10.3389/fmars.2016.00114

Novaes Filho, J. P., Selva, E. C., Couto, E. G., Lehmann, J., Johnson, M. S., & Riha., S. J. (2009). LBA-ECO ND-11 Soil Properties of Forested Headwater Catchments, Mato Grosso, Brazil. https://doi.org/10.3334/ORNLDAAC/914

Piirainen, S., Finér, L., Mannerkoski, H., & Starr, M. (2004). Effects of forest clear-cutting on the sulphur, phosphorus and base cations fluxes through podzolic soil horizons. *Biogeochemistry*, *69*(3), 405–424. https://doi.org/10.1023/B:BIOG.0000031061.80421.1b

Roose, E. (1981). *Dynamique actuelle de sols férrallitiques et férrugineux tropicaux d'Afrique Occidentale*.

Roose, E. J., & Lelong, F. (1981). Factors of the chemical composition of seepage and groundwaters in the intertropical zone (west africa). *Journal of Hydrology*, *54*, 1–22.

Schwendenmann, L., & Veldkamp, E. (2005). The role of dissolved organic carbon, dissolved organic nitrogen, and dissolved inorganic nitrogen in a tropical wet forest ecosystem. *Ecosystems*, *8*(4), 339–351. https://doi.org/10.1007/s10021-003-0088-1

SCHWENDENMANN, L., VELDKAMP, E., BRENES, T., O'BRIEN, J. J., & MACKENSEN, J. (2003). Spatial and temporal variation in soil CO2 efflux in an old-growth neotropical rain forest, La Selva, Costa Rica. *Biogeochemistry*, 1–16.

Solinger, S., Kalbitz, K., & Matzner, E. (2001). Controls on the dynamics of dissolved organic carbon and nitrogen in a Central European deciduous forest. *Biogeochemistry*, *55*(3), 327–349. https://doi.org/10.1023/A:1011848326013

Veldkamp, E., Becker, A., Schwendenmann, L., Clark, D. J. A., & Schulte-Bisping, H. (2003). Substantial labile carbon stocks and microbial activity in deeply weathered soils below a tropical wet forest. *Global Change Biology*, *9*(8), 1171–1184. https://doi.org/10.1046/j.1365-2486.2003.00656.x

Walmsley, D. C., Siemens, J., Kindler, R., Kirwan, L., Kaiser, K., Saunders, M., … Osborne, B. A. (2011). Dissolved carbon leaching from an Irish cropland soil is increased by reduced tillage and cover cropping. *Agriculture, Ecosystems and Environment*, *142*(3–4), 393–402. https://doi.org/10.1016/j.agee.2011.06.011

Yano, Y., Lajtha, K., Sollins, P., & Caldwell, B. A. (2004). Chemical and seasonal controls on the dynamics of dissolved organic matter in a coniferous old-growth stand in the Pacific Northwest, USA. *Biogeochemistry*, *71*(2), 197–223. https://doi.org/10.1007/s10533-004-8130-8

Zhao, M., Heinsch, F. A., Nemani, R. R., & Running, S. W. (2005). Improvements of the MODIS terrestrial gross and net primary production global data set. *Remote Sensing of Environment*, *95*(2), 164–176. https://doi.org/10.1016/j.rse.2004.12.011

Zhao, M., & Running, S. W. (2010). Drought-Induced Reduction in Global Terrestrial Net Primary Production from 2000 Through 2009. *Science*, *329*(5994), 940–943. https://doi.org/10.1126/science.1192666
